# Application of Epidemiological Geographic Information System: An Open-Source Spatial Analysis Tool Based on the OMOP Common Data Model

**DOI:** 10.3390/ijerph17217824

**Published:** 2020-10-26

**Authors:** Jaehyeong Cho, Seng Chan You, Seongwon Lee, DongSu Park, Bumhee Park, George Hripcsak, Rae Woong Park

**Affiliations:** 1 Department of Biomedical Sciences, Ajou University Graduate School of Medicine, Suwon 16499, Korea; boyinai03@ajou.ac.kr; 2 Department of Biomedical Informatics, Ajou University School of Medicine, Suwon 16499, Korea; chandryou@ajou.ac.kr (S.C.Y.); seongwon.lee.16@ajou.ac.kr (S.L.); dongsu2005@ajou.ac.kr (D.P.); bhpark@ajou.ac.kr (B.P.); 3 Office of Biostatistics, Ajou Research Institute for Innovative Medicine, Ajou University Medical Center, Suwon 16499, Korea; 4 Department of Biomedical Informatics, Columbia University Medical Center, New York, NY 10032, USA; gh13@cumc.columbia.edu; 5 Medical Informatics Services, New York-Presbyterian Hospital, New York, NY 10032, USA

**Keywords:** spatial epidemiology, disease clustering, geographical information system

## Abstract

Background: Spatial epidemiology is used to evaluate geographical variations and disparities in health outcomes; however, constructing geographic statistical models requires a labor-intensive process that limits the overall utility. We developed an open-source software for spatial epidemiological analysis and demonstrated its applicability and quality. Methods: Based on standardized geocode and observational health data, the Application of Epidemiological Geographic Information System (AEGIS) provides two spatial analysis methods: disease mapping and detecting clustered medical conditions and outcomes. The AEGIS assesses the geographical distribution of incidences and health outcomes in Korea and the United States, specifically incidence of cancers and their mortality rates, endemic malarial areas, and heart diseases (only the United States). Results: The AEGIS-generated spatial distribution of incident cancer in Korea was consistent with previous reports. The incidence of liver cancer in women with the highest Moran’s I (0.44; *p* < 0.001) was 17.4 (10.3–26.9). The malarial endemic cluster was identified in Paju-si, Korea (*p* < 0.001). When the AEGIS was applied to the database of the United States, a heart disease cluster was appropriately identified (*p* < 0.001). Conclusions: As an open-source, cross-country, spatial analytics solution, AEGIS may globally assess the differences in geographical distribution of health outcomes through the use of standardized geocode and observational health databases.

## 1. Introduction

A comprehensive understanding of the geospatial patterns of the incidence and outcomes of medical conditions is important for establishing hypotheses to identify disease-related factors or for planning to improve public health [1,2]. Disease mapping and clustering are promising epidemiological methodologies for evaluating the spatial distribution of diseases, both cross-sectionally and longitudinally [3,4,5,6,7,8].

Despite the accessibility of the available observational health databases and developments in spatial methodology, the required labor-intensive process often hinders active and widespread adoption of spatial analysis. In general, most efforts used to draw disease maps are aimed at acquiring geographic information system (GIS) data, preprocessing raw data, and visualizing analytical results.

To overcome the complexity of spatial analysis, we propose a standardized spatial analysis using global standardized observational health and GIS databases. This strategy enables large-scale exploration among diverse data partners, which is not limited to specific countries, and ensures transparency in research and improved reusability. In this study, we used standard medical terminology and homogeneous observational health data structures constructed through Observational Health Data Science and Informatics (OHDSI) collaborative. OHDSI is a global community of clinical application researchers, data partners, and open-source analytics tools aimed at obtaining reliable medical evidence from large-scale observational health data [9,10,11]. The OHDSI community leverages the Observational Medical Outcomes Partnership (OMOP)-Common Data Model (CDM) for standardization of data structure and semantics. Through these efforts, institutions from various countries, which have electronic health records and administrative claim data, have completed the conversion to the proposed OMOP-CDM [12]. The OHDSI claims that these are more than three billion patient data in OMOP-CDM in 152 databases from 18 countries [13].

The Global Administrative Areas (GADM) database provides high spatial resolution for all the subdivisions across 254 countries [14]. Thus, combining the OMOP-CDM and GADM databases allows the standardization of network-research-protocol sharing that can be applied to heterogeneous observational health databases among data holders across the globe.

Considering the prospects of standardizing these analysis techniques and making them available via open-source sharing, the first objective of this study was to develop an Application of Epidemiological Geographic Information System (AEGIS), an interactive tool for designing spatial epidemiological analysis based on a standardized observational health database and GIS database. The second objective of this investigation was to assess the applicability and methodological quality as a cross-national spatial analysis solution in comparison with those described by relevant published studies and reports. As proof-of-concept, we used two different reimbursement healthcare databases from South Korea and the United States to map the distribution of major cancer, malaria infections, and heart diseases (only the United States) with well-known geographical patterns of incidence, and investigate the areas of concentration.

## 2. Materials and Methods

### 2.1. AEGIS

AEGIS, an interactive spatial analytical solution based on OMOP-CDM version 5 and GADM, allows the following functions: (1) data preprocessing to prepare epidemiological analysis, including linking medical data with GIS data; (2) disease mapping function to visualize patterns of regional differences in medical events; and (3) a disease-clustering function to distinguish the geographic area where adverse outcomes occur more than expected among a group of people defined over a specific time period. In this study, R 3.3.2 (R Foundation for Statistical Computing, Vienna, Austria) and the web application framework RShiny were used to implement these functions [15,16].

### 2.2. Standardized Databases: OMOP-CDM and GADM

The OMOP-CDM consists of a common structure of patient-level data, such as personal characteristics (age, sex, and residence) and medical records (diagnoses, drugs prescribed, medical procedures, lab tests and results, and self-reported medical history). The notation of “cohorts” is often used in the OHDSI community: a concept commonly applied in observational studies using large-scale medical databases [17] and consists of patients who meet eligibility criteria related to a baseline (e.g., a person prescribed with hypertension drugs). The OHDSI ecosystem is a harmonization of these defined cohorts-based patient extraction, estimation/prediction methods of their medical outcomes, and R packages that seamlessly support this process. Cohorts can be easily defined as a phenotype via ATLAS, which is an integrated web-based and open-source software platform [18]. In addition, the cohort extraction codes defined by ATLAS are efficient and consistent because they can be transferred to other sites in a consensual format, such as various database management system queries or JavaScript Object Notation code [19].

GADM is a spatial database of administrative areas (or administrative boundaries) of 254 countries. The spatial ontology of GADM has a hierarchical system based on the administrative district system in each country from level 1 through level 6. Table 1 presents examples of this system. GADM provides high-resolution spatial information for all subdivision districts, including (1) spatial polygons (administrative boundaries), (2) the area of the administrative district, and (3) coordinates of the centroid. In this study, the GADM database, which contains geographic information for approximately all the countries in the world, was used as a standard geographic information database and reduced the time required by researchers to acquire the GIS data necessary for conducting spatial analysis.

### 2.3. Overview of the Process Model in AEGIS

AEGIS works as an end-user application of the OHDSI ecosystem. A common strategy used to convert the medical data in the OMOP-CDM into spatial analysis data is to create cohorts of patients at exposure risk (termed as the target cohort) and denoting a health outcome (termed as the outcome cohort) as well as to select an index date for each patient. The patients in the target cohort are used as the number of population in the study area (i.e., denominator). For each patient, the presence of the outcome of interest is assessed by identifying the inclusion in the outcome cohort based on the index date with respect to the risk observation period (termed as time-at-risk). In the OHDSI, cohorts are typically defined independently of the remaining cohorts in this study, allowing their reuse.

The user accesses a database obtained using OMOP-CDM selects a target and outcome from a cohort list defined and extracted from ATLAS and defines a time-at-risk. The demographic information of all the patients in the target cohort (including the sex, age on the index date, and the coordinates of the district at which they reside) was obtained from the person and location tables in OMOP-CDM. Further, the number of target patients and patients with medical outcomes are aggregated by region, sex, and age group and delivered to R based on the target/outcome cohort and time-at-risk.

**Figure 1:**
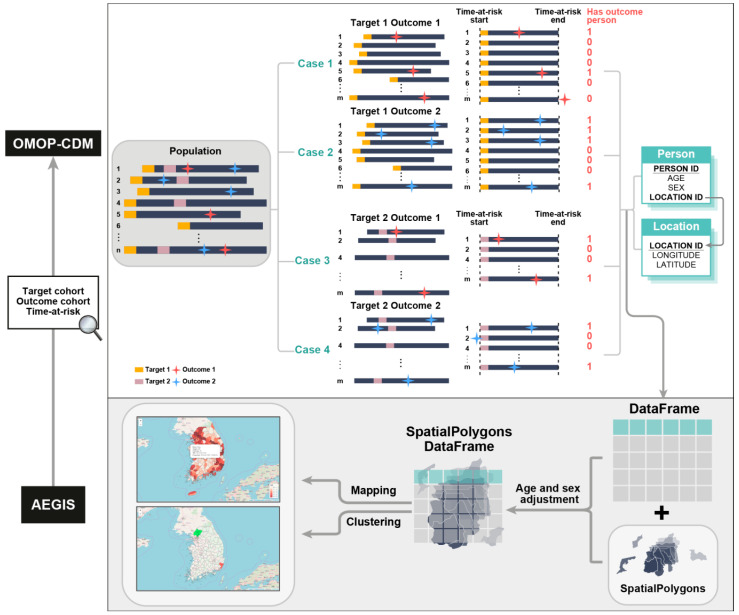
Illustration of the manner in which AEGIS based on the homogeneous structure of OMOP-CDM performs spatial epidemiology analyses. AEGIS, Application of Epidemiological Geographic Information System; OMOP-CDM, Observational Medical Outcomes Partnership-Common Data Model.

One of the features of GADM is that the administrative information is hierarchical. Thus, the number of patients in the target cohort and the number of patients who have a medical outcome are assigned to the lowest hierarchical area of GADM corresponding to the coordinates of their residential area, and diverse spatial statistics are calculated for different administration levels. Figure 1 depicts an overview of AEGIS that performs spatial epidemiology analysis using an observational health database converted to the OMOP-CDM.

### 2.4. Spatial Statistics

Disease mapping is preferred for performing public health surveillance because it allows us to explain the spatial trend associated with the high or low disease incidence, identifying areas with unusually high risk concentrations, and formulating etiology hypotheses. AEGIS maps the risk of diseases in the form of a choropleth map, with the number of patients in the user-defined target cohort, standardized incidence ratio (SIRs), proportion, and Bayesian disease mapping models (Besag–York–Mollié) [4,20]. Disease risk is estimated for each administrative subdivision divided by the observed value versus the expected value at risk area i=1⋯N. The expected counts used to estimate disease risk in each study area are calculated on the basis of the population demographics of the regions. Specifically,

(1) $$E_{i}=\overset{m}{\underset{j=1}{\sum }}r_{j}^{\left(s\right)}n_{j},$$

where $r_{j}^{\left(s\right)}$ is the rate in stratum j in case of the incidence rate on the indirect standardized population with respect to the age and sex of all the patients included in the target cohort, and $n_{j}$ is the number of target patients in stratum j of the administrative districts. In areas with small populations, proportions or SIRs can be very considerably and cannot be reliably reported owing to insufficient sample size. Conversely, Bayesian disease mapping models are preferred for obtaining improved local estimates in areas in which there is insufficient information to estimate because covariates can be incorporated by borrowing information from the surrounding area, i.e., the area bordering the area [4]. The general model for Bayesian disease mapping is expressed as follows:

(2) $$Y_{i}\sim Poisson\left(E_{i}\lambda_{i}\right),\;i=1,\cdots N,\;\log\left(\lambda_{i}\right)=X_{i}\beta +U_{i},\;U_{i}\sim BYM\left(\sigma_{1}^{2},\sigma_{2}^{2}\right),$$

where $X_{i}$ are covariates, $U_{i}$ is a spatial random effect. $\sigma_{1}^{2}$ is a spatially structured variance parameter and $\sigma_{2}^{2}$ is a spatially independent variance. Additionally, AEGIS provides adjusted regional incidence with user-defined covariates. We used the R-INLA package for Bayesian calculations for small-area estimation [20].

AEGIS also uses spatial scan statistics for detecting statistically significant disease clusters [6]. This process expands the myriad of circular scan windows in the area of interest and scans whether the outcome occurrence is more focused when compared with the outside window. For the clustering results, a *p*-value of <0.05 was considered to be statistically significant. AEGIS presents uncertainty of estimates as 95% confidence intervals (SIRs, and proportion) or credible intervals (Bayesian disease mapping model). Comprehensive information on the statistical process used here is provided in the Supplementary Materials Methods S1.

### 2.5. Data Sources

The two observational health data sources from South Korea and the United States were analyzed. National Health Insurance Service–National Sample Cohort (NHIS-NSC) is a representative cohort of South Korea, which includes longitudinal observational health data for a population of 1 million randomly sampled patients from 2002 to 2013 [21]. The NHIS-NSC includes variables that identify the characteristics of the individual, such as age, sex, income status, residential area, birth, and death. The medical treatment data include information on the medical bill, such as diagnosis (according to the ICD-10 diagnostic codes), prescription, procedure, and device that the healthcare provider has claimed.

Another data source used was the Data Entrepreneurs’ Synthetic Public Use File (DE-SynPUF) database. This database published by the Centers for Medicare Service (CMS) is a synthetic database of 5% of the US population from 2008 to 2010 based on the real Medicare claim data. The conversion process of the databases into OMOP-CDM is available at the official GitHub of OHDSI [22,23].

This study was approved and informed consent waived by the Ajou University Hospital Institutional Review Board (AJIRB-MED-EXP-18-303). All methods of this study were performed in accordance with the relevant guidelines and regulations. The study complied with the tenets of the Declaration of Helsinki.

### 2.6. Code Availability

All source codes for AEGIS are available at the OHDSI GitHub repository [24].

### 2.7. Data Availability

The data that support the findings of this study are available from NHIS and CMS [25,26]. To gain access to NHIS-NSC data, a completed application form, a research proposal, and the applicant’s institutional review board (IRB) approval document should be submitted to and reviewed by the Review Committee of Research Support in NHIS.

### 2.8. Applicability of AEGIS

For proof-of-concept, two spatial studies have been designed in South Korea and the United States with respect to the (1) geographical variation of major types of cancers and (2) identification of the endemic area of malaria. Cancer is a major non-communicable chronic disease and a leading cause of death, and its socioeconomic burden is continuously increasing [27,28,29]. Assessing the regional heterogeneity with respect to the incidence and prognosis of cancer is important to improve the public health. Malaria is another disease that is globally raising concerns with respect to public health. It is well-known that malaria exhibits an extremely distinctive regional distribution because it is transmitted by regional mosquitoes [30,31].

The target cohorts were defined as the whole population in the database, and the outcome cohort included patients with major cancers or malaria. Overall, the following seven major cancers were classified by using the International Classification of Diseases, 10th Revision (ICD-10) diagnostic codes for analysis of the 5-year periods of 2004–2008 and 2009–2013: stomach (C16), colorectal (C18–C20), liver (C22), lung (C33–C34), breast (C50), prostate (C61), and thyroid (C73). Additionally, the spatial distribution of 5-year mortality in patients with incident cancers between 2004 and 2008 was described. Malaria in this study was defined between 2008 and 2010 according to the ICD-10: B50–B53.

In the study area, the incidence and mortality rates of the disease were estimated using the Bayesian disease mapping model, taking into account the small areas with insufficient samples. In addition, scan statistics were used to identify the geospatial clusters of the hot spots after adjustment for age and sex. The output of AEGIS, a choropleth map, identifies geographical changes and trends in major cancer incidence and mortality rates and identifies regions where malaria patients are concentrated in Korea and the United States. In this regard, Moran’s I statistic was calculated to evaluate local heterogeneity. Moran’s I represents the overall spatial autocorrelation of the area covered by the study. The values range from −1 (indicating dispersed distribution) to 1 (perfect clustering). A value of zero indicates no autocorrelation [3].

Since the United States is one of the countries where malaria is not endemic or is no longer being transmitted [30], it can lead to the failure to detect statistically significant clusters. These results cannot be considered suitable for the verification of AEGIS. Therefore, additional analysis was conducted to further compare the regional distribution of hospitalizations due to heart disease previously published from the United States Centers for Disease Control and Prevention and to detect clusters in the results. We studied the following 6 outcomes of interest: stroke, acute myocardial infarction, cardiac dysrhythmia, coronary heart disease, heart failure, and heart disease [32].

### 2.9. Verification of Methodological Quality of AEGIS

To validate the spatial analytical function of AEGIS, outputs were compared with relevant published reports and studies. First, we compared the annual national incidence (cases per 100,000 persons) of seven major cancers (stomach, colorectal, liver, lung, breast, prostate, and thyroid) in Korea calculated by using AEGIS with the data reported by Statistics Korea [33] and determined whether it overlapped within the 95% credible interval. The Korea Central Cancer Registry is a database that collects nationwide hospital-based cancer incidence data since 1980 and annually provides incidence, survival, and prevalence statistics [34]. To assess the credibility of the clustering findings generated from AEGIS, the geographical cluster of malaria in South Korea was compared with that of the previously published studies [35,36,37].

## 3. Results

### 3.1. Graphical User Interface of AEGIS

**Figure 2:**
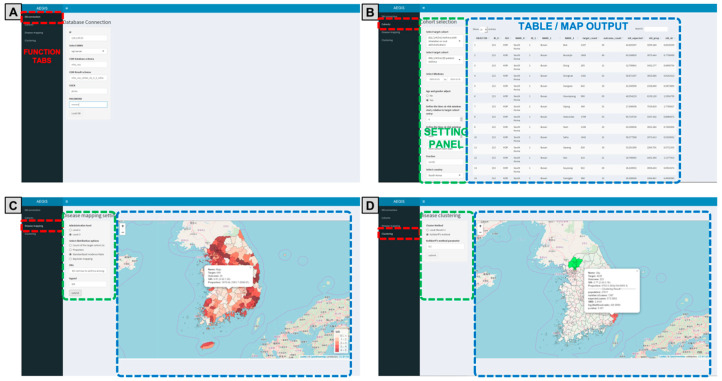
Graphical user interface of AEGIS. Four function tabs (red box), setting panel (green box), and table and map outputs (blue box). AEGIS, Application of Epidemiological Geographic Information System. (**A**) DB connection: A panel to set the server address, username, password, database management system, and database schema to configure the OMOP-CDM server connection; (**B**) Cohorts: Select user parameters for processing specific data, such as target/outcome cohort, age and gender adjustment, time at risk and country; (**C**) Disease mapping: Output disease mapping results based on user parameters designed in the Cohorts panel; (**D**) Visualize the results according to the selected clustering method.

The AEGIS graphical user interface is classified into four functional panels (Figure 2, Supplementary Materials Information S1). The database connection panel features an input box for entering the server information to connect with the CDM database. The option buttons in the cohorts panel are used for handling specific data (i.e., age and gender adjustment, time at risk, and country selection), and the output for displaying the results table is provided. Finally, the disease mapping and cluster panels provide option boxes for selecting analytical methods and an input box for entering parameters. The output space is utilized to visualize the analytical results. The output of the disease map provides interactive panning and zooming as well as clicking on the administrative polygon to supply information, such as the area name, target/outcome count, and risk of disease with 95% confidence or credible interval. The source code is available at the official GitHub of OHDSI [24], which provides free access and encourages user contributions.

### 3.2. Geographical Distribution of Major Cancers

**Figure 3:**
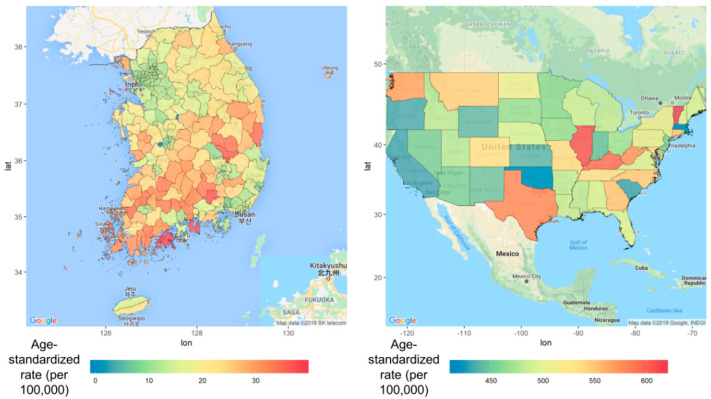
Disease mapping for regional differences in the incidence of liver cancer (women) in South Korea and the United States from 2009 to 2013 (age adjusted).

Table 2 shows the statistical significance of Moran’s I calculations for the incidence of major cancers, including liver, lung, stomach, thyroid, breast, and prostate (positive values for Moran’s I; all *p*-values <0.05), but not statistically significant in the colorectal and breast cancer. Figure 3 shows the geographic variation in the incidence of female liver cancer from 2009 to 2013, the cancer with the highest Moran’s I value (0.44; *p* < 0.001). The incidence rate of female liver cancer was typically higher on the southwest coast and southeast inland.

In the United States, Vermont (=614.98 [429.23–949.51]) was the region with the highest incidence of female liver cancer per 100,000 women per year, followed by Illinois (=607 [477.39–774.78]) and Kentucky (=595 [432.02–851.66]) (Figure 3). In addition, Texas (=580 [471.01–716.27]) in the southern region also had a higher incidence of liver cancer in women. Details of other cancers’ incidence and mortality rates are shown in Supplementary Materials Figure S1.

Table 2 shows also that Moran’s I calculated to identify regional disparities in major cancer mortalities was not statistically significant (including colorectal, liver [only women], lung [only men], stomach, breast, and prostate cancer; all *p*-values ≥0.05). The difference in the incidence of lung cancer between women and men (Moran’s I, −0.18; *p* < 0.001) and men (Moran’s I, −0.05; *p* < 0.001) was statistically significant, but Moran’s I was a negative value.

All cancer incidences reported by Statistics Korea [33] are within the 95% credible interval range of all cancer incidences estimated by AEGIS (Table 3). The increasing or decreasing temporal trends in the incidences of cancers assessed by AEGIS were also similar to those of previous reports (Supplementary Materials Figure S2). Further analysis was performed by comparing with another publication [38] with respect to the national major cancer incidence during the same period in Korea to denote that the AEGIS analysis results are robust (Supplementary Materials Table S1).

### 3.3. Identification of Endemic Areas of Malaria

**Figure 4:**
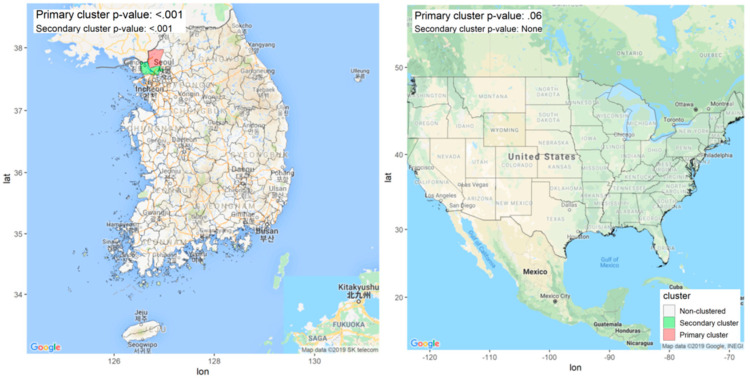
Malaria high-incidence clusters in South Korea and the United States from 2008 to 2010 (age and sex adjusted).

Malaria epidemics in South Korea are highly concentrated near the military demarcation line (especially, in the northern areas), which forms the boundary with North Korea (Figure 4). As shown in Figure 4, the primary cluster of malaria was detected in the GADM level 3 administrative area of Paju-si (*p* < 0.001), and the secondary cluster was identified in the GADM level 3 administrative areas of Gimpo-si and Goyang-si (*p* < 0.001). We found that AEGIS was able to describe geographic distribution and identify the known primary cluster of malaria in case of Paju-si [35,36,37]. The main reason for this phenomenon is consistent with the opinion that the infective mosquitoes of North Korea flowed into South Korea through the western part of the military demarcation line [39,40]. Furthermore, the incidence of malaria in North Korea was also the highest near the military demarcation line [30,37].

**Figure 5:**
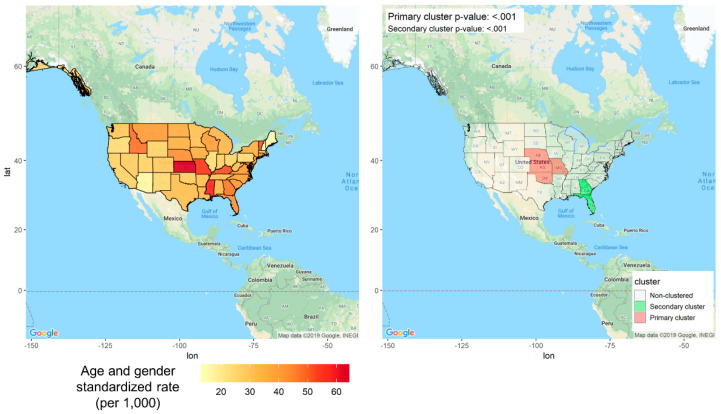
Disease mapping and clustering for regional differences in the incidence of all heart diseases in the United States from 2008 to 2010 (age and gender adjusted).

Figure 4 denotes the results of identifying geographic clusters in the United States. The scan statistics for identifying geographical clusters were the highest in Arizona but; the difference was not statistically significant (*p* = 0.06). The overall incidence of malaria infections in the United States is extremely rare and not concentrated in certain areas. Most cases of malaria infections can be observed among people who traveled to countries with an ongoing malaria epidemic rather than for endemic reasons [41]. Therefore, regional incidence distribution mapping and cluster detection were performed for the six outcomes of interest in the United States: stroke, acute myocardial infarction, cardiac dysrhythmia, coronary heart disease, heart failure, and heart disease. The geographical distribution of hospitalization rates was higher than in other regions in the southeastern region called the “stroke belt” [42,43]. Statistically significant clusters were found for all heart disease, all strokes, cardiac dysfunction, coronary heart disease, and heart failure (all *p*-values <0.05). As shown in Figure 5, Kansas (=63.20 [55.27–71.62]) was the highest annual incidence of all heart disease per 1000 people, and primary cluster including Nebraska, Missouri, and Oklahoma was identified, centering on Kansas (*p* < 0.001). See Supplementary Materials Figure S3 for detailed information about the geographical distribution of hospitalization due to heart disease in the United States.

## 4. Discussion

In this study, we developed an open-source software application named AEGIS that allows investigators to explore the spatial distribution of user-defined medical events and identify geographic clusters with unusually better or worse health outcomes according to the various regional granularities based on global standardized geocode and an observational health database. In particular, the inherent aspects of GIS are completely implemented in AEGIS to enable the inferring adjacency and computing distance for small-area estimation and scan statistics. The GADM would have uniform and reliable geographic information at common levels of resolution for virtually all countries, while observational health databases converted to OMOP-CDM would have widely-accepted terminologies and taxonomies accompanied using widespread compliance and contributions. The feasibility and reliability of AEGIS were demonstrated in multiple proof-of-concept studies using databases from two countries with different administrative districts. AEGIS generated accurate geographical distribution of the medical conditions and outcomes when compared with the findings of previous reports. It was possible to easily identify endemic areas of malaria and assess geospatial heterogeneity in five-year mortality rates of cancer patients.

Efforts such as geospatial open-source projects (e.g., Open GIS Consortium, Humanitarian OpenStreetMap Team, and Health Atlas by Institute for Health Metrics and Evaluation), the GIS software (mapping tools from international GIS companies), and research studies [20,44,45] that aimed at improving public health have been made for decades. In particular, SpatialEpiApp reported by Moraga and STVCapp by Chao et al. have many similarities to AEGIS in supporting advanced spatial modeling and mapping for health-related data [45,46,47,48]. In contrast, AEGIS is a series of processing models, including the standardization of the global administrative geocodes for observational health databases and modules to represent geographical patterns, such as epidemiological indicators and visualization, and the application of spatial analysis methods based on open-source ecosystems. Since the AEGIS was also developed based on standardized data structures and terminologies, it ensures reusability, interoperability, and transparency of the research results. Validation of the AEGIS was conducted comprehensively. The AEGIS-generated consistent reports on spatial distribution of major cancers when compared with the incidences reported by Statistics Korea based on the National Cancer Registry [33].

The AEGIS allows public health investigators to monitor areas of unusual disease occurrence, analyze areas in which health problems are concentrated, and visualize results without programming skills for spatial epidemiology. From a geoscience perspective, AEGIS-generated figures and analyses are common, but identifying regional differences in health outcomes and reducing regional health disparities are important in promoting public health. The visual representation of the distribution of county or even finer scales on the spatial trends of major cancer incidences enabled an investigation of the social inequality as associated with outcomes. In an autocorrelation analysis to assess the regional disparities in the incidence and mortality rates of major cancers, the incidence rate of liver cancer among women was found to be highest between 2009 and 2013. This regional pattern of a high female liver cancer incidence rate concentrated along the southwest coast and in the southeast inland areas might be related to a similar regional pattern of well-known risk factors for liver cancer, such as heavy alcohol intake, parasitic infections, or viral hepatitis, that were previously found to be high [49,50,51]. The geospatial distribution of five-year mortality rate among patients with major cancer was either not autocorrelated or rather dispersed. This finding suggests that equitable healthcare services at the national level were provided to those patients, which could be due to the high level of healthcare accessibility and quality in South Korea [52]. In-depth assessments and additional studies should be performed to authenticate this finding.

It is possible to establish standardization for incorporating administrative area-based materials and to estimate the association between various environmental factors (e.g., air pollution and socioeconomic and genetic factors) and occurrence of medical events, such as cancers, communicable diseases, revascularization, and asthma-related hospitalizations. Furthermore, standardized environmental factors can be used as covariates in the Bayesian disease mapping model supported by the AEGIS, which indicates that it is straightforward to use them as a starting point for complex epidemiological studies to evaluate and compare related environmental factors between diseases. All source codes for the AEGIS are publicly available. Therefore, any data holder with observational health data in the OMOP-CDM can freely use the AEGIS. To improve the AEGIS, its code can be modified using suggestions of researchers from many fields, such as spatial epidemiology or OHDSI. To our knowledge, the AEGIS is the first open-source software that allows flexible spatial analyzation and visualization of user-defined medical cohorts based on a global standardized geocode and observational health database.

This study exhibits some limitations that should be addressed. First, observational health data should be standardized to OMOP-CDM for use in AEGIS. However, the OHDSI community is expanding rapidly, and collaborators have already converted many observational health databases into OMOP-CDM, or there are literature and tools to facilitate this. Second, AEGIS does not provide latitude and longitude coordinate-based analysis because we wished to protect sensitive personal information. Third, disease surveillance through disease clustering can generate false alarms (false positives). Therefore, attention is needed when interpreting whether the trends and clusters found are caused by deterministic and/or randomly components. AEGIS provides a Bayesian mapping model to estimate adjusted regional risks using user-defined covariates. Fourth, only Korean and United States data were used for the investigation conducted in this study. However, the United States data used in this study are synthetic; therefore, the results should be carefully interpreted. Thus, AEGIS should be validated further by using additional real-world databases from other countries, including the United States.

## 5. Conclusions

In summary, AEGIS is an interactive open-source software management system that allows researchers to perform spatial analysis in epidemiology and achieve visualized results from observational health databases in the OMOP-CDM. We demonstrated the applicability and methodological quality validation of AEGIS across countries by using proof-of-concept studies. AEGIS may expedite research on public health by providing quick insights into regional disparities in medical conditions. AEGIS and its source codes are freely available at GitHub [24].

## Figures and Tables

**Table 1:** Examples of the subdivision of administrative districts in South Korea, the United States, and the Netherlands provided by the Global Administrative Areas database.

	South Korea	United States	Netherlands
Level 1: nation	South Korea	United States	Netherlands
Level 2: states	Seoul	Illinois	South Holland
Level 3: county	Gangnam-gu	Springfield	Rotterdam

**Table 2:** Results of Moran’s I statistical test for the incidence and mortality of cancers in South Korea.

Cancer Site		Incidence				Mortality	
		2004–2008		2009–2013		2004–2008	
		Moran’s I	*p*-Value	Moran’s I	*p*-Value	Moran’s I	*p*-Value
Colorectal	Men	0.08	0.17	0.05	0.23	−0.06	0.23
	Women	−0.01	0.95	0.03	0.56	−0.06	0.56
Liver	Men	0.37	<0.001	0.39	<0.001	−0.05	0.05
	Women	0.42	<0.001	0.44	<0.001	−0.18	<0.001
Lung	Men	0.34	<0.001	0.34	<0.001	−0.05	<0.001
	Women	0.38	<0.001	0.40	<0.001	−0.08	0.58
Stomach	Men	0.33	<0.001	0.32	<0.001	−0.05	0.57
	Women	0.40	<0.001	0.39	<0.001	−0.05	0.45
Thyroid	Men	0.40	<0.001	0.40	<0.001	-	-
	Women	0.29	<0.001	0.30	<0.001	-	-
Breast	Men	-	-	-	-	-	-
	Women	0.36	<0.001	0.36	0.08	−0.09	0.05
Prostate	Men	0.36	<0.001	0.39	<0.001	−0.08	0.05
	Women	-	-	-	-	-	-

**Table 3:** Comparison of the estimated major cancer incidences (age adjusted) from AEGIS with the findings of relevant published reports.

Cancer Site		National Incidences (Cases Per 100,000 Persons)			
		2004–2008		2009–2013	
		AEGIS	Statistics Korea ^1^	AEGIS	Statistics Korea ^2^
Colorectal	Men	47.2 (31.8–66.6)	47.6	61.8 (41.7–86.9)	69.5
	Women	33.3 (22.2–47.2)	33.7	44.5 (29.1–64.4)	44.5
Liver	Men	48.9 (31.3–72.3)	45.9	46.4 (32.6–63.8)	49.3
	Women	17.4 (10.3–26.9)	15.4	18.5 (12.2–26.2)	17.4
Lung	Men	59.6 (42–81.8)	51.7	62.9 (47.5–80.6)	61.5
	Women	20.5 (13.5–28.9)	20.7	25.1 (13.6–41.4)	26.9
Stomach	Men	67.4 (47.8–91.8)	72.4	73.4 (52.5–99.2)	85.8
	Women	33.6 (23.9–45.4)	35.7	34.1 (26.9–42)	41.6
Thyroid	Men	8.6 (3.1–18.5)	9.5	24.8 (12.2–43.9)	28.3
	Women	52.1 (29.6–83.8)	56.6	104.9 (65.8–156.9)	136.4
Breast	Men	-	-	-	-
	Women	33.2 (23.3–45.0)	44.6	44.9 (26.1–70.9)	64.3
Prostate	Men	20.7 (11–34.6)	18.4	28.0 (15.3–45.9)	36.2
	Women	-	-	-	-

^1^ 2006 cancer incidence (statistics Korea); ^2^ 2011 cancer incidence (statistics Korea).

## Supplementary Materials

The following are available online at https://www.mdpi.com/1660-4601/17/21/7824/s1, Methods S1: Statistical method in AEGIS. Information S1: Interactive web application. Table S1: Comparison of the estimated major cancer incidences (age adjusted) from AEGIS with the findings of relevant published reports. Figure S1: County-level age-adjusted geographical variation in incidence and mortality of major cancers in Korea. Figure S2: Comparison of GADM-level 2 major cancer age-standardized incidence rate from AEGIS and major cancer age-standardized incidence rate from NCC in Korea. Figure S3: Disease mapping and clustering for regional differences in hospitalization rates due to heart-related diseases per 1000 people in the United States from 2008 to 2010 (age and sex adjusted).

## References

[1] Lansdorp-Vogelaar I., Goede S.L., Ma J., Xiau-Cheng W., Pawlish K., Van Ballegooijen M., Jemal A. (2015). State disparities in colorectal cancer rates: Contributions of risk factors, screening, and survival differences. Cancer.

[2] Dwyer-Lindgren L., Bertozzi-Villa A., Stubbs R.W., Morozoff C., Kutz M.J., Huynh C., Barber R.M., Shackelford K.A., Mackenbach J.P., Van Lenthe F.J. (2016). US county-level trends in mortality rates for major causes of death, 1980–2014. JAMA J. Am. Med. Assoc..

[3] Cliff A.D., Ord J.K. (1973). Spatial Autocorrelation.

[4] Besag J. (1991). Rejoinder. Ann. Inst. Stat. Math..

[5] Anselin L. (1995). Local Indicators of Spatial Association—LISA. Geogr. Anal..

[6] Kulldorff M. (1997). A spatial scan statistic. Commun. Stat. Methods.

[7] Elliott P., Wartenberg D. (2004). Spatial epidemiology: Current approaches and future challenges. Environ. Health Perspect..

[8] Kihal W., Padilla C., Deguen S. (2017). The need for, and value of, a spatial scan statistical tool for tackling social health inequalities. Glob. Health Promot..

[9] Hripcsak G., Duke J.D., Shah N.H., Reich C.G., Huser V., Schuemie M.J., Suchard M.A., Park R.W., Wong I.C.K., Rijnbeek P.R. (2015). Observational Health Data Sciences and Informatics (OHDSI): Opportunities for Observational Researchers. Stud. Health Technol. Inform..

[10] Hripcsak G., Ryan P.B., Duke J.D., Shah N.H., Park R.W., Huser V., Suchard M.A., Schuemie M.J., DeFalco F.J., Perotte A. (2016). Characterizing treatment pathways at scale using the OHDSI network. Proc. Natl. Acad. Sci. USA.

[11] Home OHDSI/CommonDataModel Wiki. https://github.com/ohdsi/commondatamodel/wiki.

[12] You S.C., Lee S., Cho S.Y., Park H., Jung S., Cho J., Yoon D., Park R.W. (2017). Conversion of National Health Insurance Service-National Sample Cohort (NHIS-NSC) Database into Observational Medical Outcomes Partnership-Common Data Model (OMOP-CDM). Stud. Health Technol. Inform..

[13] Resources:2019_data_network [Observational Health Data Sciences and Informatics]. https://www.ohdsi.org/web/wiki/doku.php?id=resources:2019_data_network.

[14] GADM (2018). GADM Database of Global Administrative Areas-Version 2.8. http://www.gadm.org.

[15] Team R.C. (2013). R: A Language and Environment for Statistical Computing. https://cran.r-project.org/.

[16] Chang W., Cheng J., Allaire J., Xie Y., McPherson J. Shiny: Web application framework for R. R Package Version 2017. https://cran.r-project.org/web/packages/shiny/index.html.

[17] Hernán M.A., Robins J.M. (2016). Using Big Data to Emulate a Target Trial When a Randomized Trial Is Not Available. Am. J. Epidemiol..

[18] Documentation: Software: Atlas [Observational Health Data Sciences and Informatics]. http://www.ohdsi.org/web/wiki/doku.php?id=documentation:software:atlas.

[19] Hripcsak G., Shang N., Peissig P.L., Rasmussen L.V., Liu C., Benoit B., Carroll R.J., Carrell D.S., Denny J.C., Dikilitas O. (2019). Facilitating phenotype transfer using a common data model. J. Biomed. Inform..

[20] Lindgren F., Rue H. (2015). Bayesian Spatial Modelling with R-INLA. J. Stat. Softw..

[21] Lee J., Lee J.S., Park S.H., Shin S.A., Kim K.W. (2017). Cohort Profile: The National Health Insurance Service-National Sample Cohort (NHIS-NSC), South Korea. Int. J. Epidemiol..

[22] ETL-NHIS_NSC. https://github.com/OHDSI/ETL---Korean-NSC.

[23] OHDSI/ETL-CMS: Workproducts to ETL CMS Datasets into OMOP Common Data Model. https://github.com/OHDSI/ETL-CMS.

[24] Jaehyeong C. Application for Epidemiological Geographic Information System (AEGIS): An Open Source Spatial Analysis Tool Based on CDM. https://github.com/ohdsi/aegis.

[25] National Health Insurance Data Sharing Service. https://nhiss.nhis.or.kr/bd/ab/bdaba000eng.do;jsessionid=FO28A6WJMpDaJqefHZmShBa1a4Eywx8xeH0RGFasM8AFME8j4nIHEWsgW3KwgfG1.primrose22_servlet_engine1.

[26] CMS 2008-2010 Data Entrepreneurs’ Synthetic Public Use File (DE-SynPUF) | CMS. https://www.cms.gov/Research-Statistics-Data-and-Systems/Downloadable-Public-Use-Files/SynPUFs/DE_Syn_PUF.

[27] Roth G.A., Abate D., Abate K.H., Abay S.M., Abbafati C., Abbasi N., Abbastabar H., Abd-Allah F., Abdela J., Abdelalim A. (2018). Global, regional, and national age-sex-specific mortality for 282 causes of death in 195 countries and territories, 1980–2017: A systematic analysis for the Global Burden of Disease Study 2017. Lancet.

[28] Yabroff K.R., Lund J., Kepka D., Mariotto A. (2011). Economic Burden of Cancer in the US. Cancer Epidemiol. Biomark. Prev..

[29] Lee K.S., Chang H.S., Lee S.M., Park E.C. (2015). Economic burden of cancer in Korea during 2000–2010. Cancer Res. Treat..

[30] World Health Organization (2016). World Malaria Report 2015.

[31] Autino B., Noris A., Russo R., Castelli F. (2012). Epidemiology of malaria in endemic areas. Mediterr. J. Hematol. Infect. Dis..

[32] Centers for Disease Control and Prevention (2014). Interactive Atlas of Heart Disease and Stroke.

[33] KOSIS KOrean Statistical Information Service. http://kosis.kr/eng/.

[34] Jung K.W., Won Y.J., Kong H.J., Lee E.S., Kim C.H., Yoo C.I., Kim Y.D., Nam H.S., Huh J.S., Youm J.H. (2019). Cancer statistics in Korea: Incidence, mortality, survival, and prevalence in 2016. Cancer Res. Treat..

[35] Moon J.J., Cho S.Y. (2001). Incidence patterns of vivax malaria in civilians residing in a high-risk county of Kyonggi-do (province), Korea. Korean J. Parasitol..

[36] Hwang S.M., Yoon S.J., Jung Y.M., Kwon G.Y., Jo S.N., Jang E.J., Kwon M.O. (2016). Assessing the impact of meteorological factors on malaria patients in demilitarized zones in Republic of Korea. Infect. Dis. Poverty.

[37] Battle K.E., Lucas T.C.D., Nguyen M., Howes R.E., Nandi A.K., Twohig K.A., Pfeffer D.A., Cameron E., Rao P.C., Casey D. (2019). Mapping the global endemicity and clinical burden of Plasmodium vivax, 2000–2017: A spatial and temporal modelling study. Lancet.

[38] Won Y., Jung K., Oh C., Kong H., Lee D.H., Lee K.H. (2018). Geographical Variations and Trends in Major Cancer Incidences throughout Korea during 1999–2013. Cancer Res. Treat. Off. J. Korean Cancer Assoc..

[39] Chai J.Y. (1999). Re-emerging Plasmodium vivax malaria in the Republic of Korea. Korean J. Parasitol..

[40] Han E.T., Lee D.H., Park K.D., Seok W.S., Kim Y.S., Tsuboi T., Shin E.H., Chai J.Y. (2006). Reemerging vivax malaria: Changing patterns of annual incidence and control programs in the Republic of Korea. Korean J. Parasitol..

[41] Mace K.E., Arguin P.M., Lucchi N.W., Tan K.R. (2019). Malaria Surveillance—United States, 2016. MMWR. Surveill. Summ..

[42] Lanska D.J., Kryscio R. (1994). Geographic distribution of hospitalization rates, case fatality, and mortality from stroke in the United States. Neurology.

[43] El-Saed A., Kuller L.H., Newman A.B., Lopez O., Costantino J., McTigue K., Cushman M., Kronmal R. (2006). Geographic variations in stroke incidence and mortality among older populations in four US communities. Stroke.

[44] Reis B.Y., Kirby C., Hadden L.E., Olson K., McMurry A.J., Daniel J.B., Mandl K.D. (2007). AEGIS: A robust and scalable real-time public health surveillance system. J. Am. Med. Inform. Assoc..

[45] Moraga P. (2017). SpatialEpiApp: A Shiny web application for the analysis of spatial and spatio-temporal disease data. Spat. Spatiotemporal. Epidemiol..

[46] Song C., Shi X., Bo Y., Wang J., Wang Y., Huang D. (2019). Exploring spatiotemporal nonstationary effects of climate factors on hand, foot, and mouth disease using Bayesian Spatiotemporally Varying Coefficients (STVC) model in Sichuan, China. Sci. Total Environ..

[47] Song C., Shi X., Wang J. (2020). Spatiotemporally Varying Coefficients (STVC) model: A Bayesian local regression to detect spatial and temporal nonstationarity in variables relationships. Ann. GIS.

[48] STVCapp. https://chaosong.shinyapps.io/stvcapp/.

[49] Kim D.Y., Kim I.H., Jeong S.H., Cho Y.K., Lee J.H., Jin Y.J., Lee D., Suh D.J., Han K.H., Park N.H. (2013). A nationwide seroepidemiology of hepatitis C virus infection in South Korea. Liver Int..

[50] Moon S., Han J.H., Bae G.R., Cho E., Kim B. (2016). Hepatitis A in Korea from 2011 to 2013: Current epidemiologic status and regional distribution. J. Korean Med. Sci..

[51] Woon Kwak C., Han K.T., Mo Nam C., Tae Moon K., Yoon H.S., Park E.C. (2017). Income disparity and mortality among patients with alcohol use disorder in South Korea. Psychiatry Res..

[52] Barber R.M., Fullman N., Sorensen R.J.D., Bollyky T., McKee M., Nolte E., Abajobir A.A., Abate K.H., Abbafati C., Abbas K.M. (2017). Healthcare Access and Quality Index based on mortality from causes amenable to personal health care in 195 countries and territories, 1990–2015: A novel analysis from the Global Burden of Disease Study 2015. Lancet.

