# Supplementary material for: Application of Epidemiological Geographic Information System: An Open-Source Spatial Analysis Tool Based on the OMOP Common Data Model
Source: Int J Environ Res Public Health. 2020 Oct 26;17(21):7824. doi: 10.3390/ijerph17217824 (PMC7663469; doi:10.3390/ijerph17217824)
Supplement: Supplementary file 1 [file ijerph-17-07824-s001.pdf]

Supplementary Materials

Application of Epidemiological Geographic Information System: An Open-Source Spatial Analysis Tool Based on the OMOP Common Data Model

Jaehyeong Cho[1,*]; Seng Chan You, MD, MS[2,*]; Seongwon Lee, PhD[2]; DongSu Park[2]; Bumhee Park, PhD[2,3]; George Hripcsak, MD, MS[4,5]; Rae Woong Park, MD, PhD[1,2†]

[1]Department of Biomedical Sciences, Ajou University Graduate School of Medicine, Suwon, Korea;
[2]Department of Biomedical Informatics, Ajou University School of Medicine, Suwon, Korea;
[3]Office of Biostatistics, Ajou Research Institute for Innovative Medicine, Ajou University Medical Center, Suwon, Republic of Korea;
[4]Department of Biomedical Informatics, Columbia University Medical Center, New York, NY USA;
[5]Medical Informatics Services, NewYork-Presbyterian Hospital, New York, NY USA
*These authors contributed equally to this work
†Address for Correspondence:
Rae Woong Park, MD, PhD
Department of Biomedical Informatics, Ajou University School of Medicine, 164 World cup-ro, Yeongtong-gu, Suwon-si, Gyeonggi-do 16499, Republic of Korea
Tel: +82-31-219-4471
E-mail: veritas@ajou.ac.kr

Supplementary Methods S1: Statistical method in AEGIS

Expected count

In AEGIS, the expected count is used several statistical calculations. Specifically,

$$E_i = \sum_{j=1}^{m} r_j^{(s)} n_j, \quad (1)$$

$r_j^{(s)}$ is the rate in stratum j in the incidence rate on indirect standardized population for age and sex of all patients included in the target cohort

n_j is the population in stratum j of the administrative district.

Standardized Incidence Ratio (SIR)

The disease risk is estimated by the SIR, which is calculated as the ratio of the observed number of outcomes to the expected counts:

$$SIR_i = \frac{Y_i}{E_i}, \quad (2)$$

Y_i is the case of the outcome in administrative district $\mathrm{i} = 1 \cdots \mathrm{N}$

E_i is expected counts of the outcome in administrative district.

Proportion

The proportion indicates the number of patient outcomes per n population in the administrative district. The fraction is used as a parameter to represent the value of the proportion.

$$Proportion_i = \left(\frac{Y_i}{\sum_{j=1}^{m} n_j} SIR_i\right) fraction \quad (3)$$

Scan Statistics

AEGIS supports to identify clusters by Kulldorff's scan statistics. This method scans an unusual number of cases, expanding a myriad of windows in the area of interest. Generally, a circle that does not contain more than 50% of the total number of patients in the target cohorts is detected using a window whose radius increases continuously from zero to the upper limit. Then, a likelihood ratio test is used to assess the occurrence of clusters statistically. The scan statistics method can assess disease occurrences and social inequalities in health quantitatively by detecting areas where adverse outcomes are concentrated. For the clustering results, *p*-value <0.05 was considered significant. Conditioning on the observed total number of outcomes C, the likelihood ratio of S is the expressed as

$$\mathrm{S} = max_Z \frac{L(Z)}{L_0}, \quad (4)$$

Z is the overall window

$\mathrm{L(Z)}$ is the likelihood for window Z

L_0 is a likelihood function under the null hypothesis that the probability inside Z is the same as the probability outside Z

$$\frac{L(Z)}{L_0} = \left(\frac{c}{E(Z)}\right)^c \left(\frac{C-c}{C-E(Z)}\right)^{C-c},$$

Bayesian mapping

To estimate disease risk in relatively poorly informed areas, the AEGIS uses the Besag-York-Molli é (BYM) model, which is a well-established method to estimate areas with a small sample size. Specifically,

$$Y_i \sim Poisson(E_i \lambda_i), i = 1, \cdots N;$$
$$\log(\lambda_i) = X_i \beta + U_i$$
$$U_i \sim BYM(\sigma_1^2, \sigma_2^2),$$

X_i are covariates

U_i is a spatial random effect. σ_1^2 is a spatially structured variance parameter and σ_2^2 is a spatially independent variance.

The R-INLA package was used for Bayesian calculations for small area estimation.

Global Moran's I

Global Moran's I representing the overall spatial autocorrelation of the area covered by the study. The values range from −1 (indicating dispersed distribution) to 1 (perfect clustering together). A value of 0 for indicates no autocorrelation. Global Moran's I is the expressed as

$$\mathrm{I} = \frac{n \sum_{i=1}^{n} \sum_{j=1}^{n} \omega_{ij} (x_i - \bar{x})(x_j - \bar{x})}{\left(\sum_{i=1}^{n} \sum_{j=1}^{n} \omega_{ij}\right) \sum_{i=1}^{n} (x_i - \bar{x})^2}$$

x_i is the attribute value of the I'th object

n is the number of objects

ω_{ij} is the weight of the commination i, j

Local Moran's $\mathbf{I}$

AEGIS calculates regional autocorrelation between individual regions using the Local Indicators of Spatial Association (LISA) method to identify clusters Specifically,.

$$I_i = \frac{(x_i - \bar{x})}{S_x} \sum_{j \neq i}^{n} \omega_{ij} \frac{(x_j - \bar{x})}{S_x},$$

$$S_x = \sqrt{\frac{\sum_{i=1}^{n} (x_i - \bar{x})^2}{n-1}},$$

n is represents the number of unit areas

where, ω_{ij} is a spatial weight for judging whether 'spatial adjacency' is in the unit area i and j, and a space weight value is given based on whether the boundary between the two unit areas is shared. In other words, $\omega_{ij} = 1$ if unit i and j share a boundary, otherwise $\omega_{ij} = 0$.

Supplementary Table S1. Comparison of the estimated major cancer incidences (age adjusted) from AEGIS with the findings of relevant published reports.

Cancer site	Classification	National incidences (cases per 100,000 persons)					
		2004–2008			2009–2013		
		AEGIS	Statistics Korea[a]	Won et al.	AEGIS	Statistics Korea[b]	Won et al.
**Colorectal**							
	Men	47.2 [31.8−66.6]	47.6	44.3	61.8 [41.7−86.9]	69.5	50.8
	Women	33.3 [22.2−47.2]	33.7	24.8	44.5 [29.1−64.4]	44.5	27.4
**Liver**							
	Men	48.9 [31.3−72.3]	45.9	42.3	46.4 [32.6−63.8]	49.3	36.8
	Women	17.4 [10.3−26.9]	15.4	11.4	18.5 [12.2−26.2]	17.4	10.2
**Lung**							
	Men	59.6 [42−81.8]	51.7	50.1	62.9 [47.5−80.6]	61.5	46.6
	Women	20.5 [13.5−28.9]	20.7	14.3	25.1 [13.6−41.4]	26.9	15.4
**Stomach**							
	Men	67.4 [47.8−91.8]	72.4	66.5	73.4 [52.5−99.2]	85.8	63.0
	Women	33.6 [23.9−45.4]	35.7	27.3	34.1 [26.9−42]	41.6	26.3
**Thyroid**							
	Men	8.6 [3.1−18.5]	9.5	9.8	24.8 [12.2−43.9]	28.3	24.3
	Women	52.1 [29.6−83.8]	56.6	55.2	104.9 [65.8−156.9)]	136.4	110.6
**Breast**							
	Men	-	-		-	-	
	Women	33.2 [23.3−45.0]	44.6	39.0	44.9 [26.1−70.9]	64.3	49.5
**Prostate**							
	Men	20.7 [11−34.6]	18.4	19.0	28.0 [15.3−45.9]	36.2	26.5
	Women	-	-		-	-	

All cancer incidence reported by Statistics Korea is within the 95% CI range of all cancer incidence estimated by AEGIS. In addition, the trend of increasing or decreasing the incidence of cancer between the two periods (2004–2008 and 2009–2013) except male liver cancer incidence is the same as the one estimated by AEGIS and the one published by Statistics Korea. Geographical variations of major cancer incidence generated by AEGIS were similar to regional cancer incidence rates reported by the Statistics Korea and Won et al (*Won Y, Jung K, Oh C, Kong H, Lee DH, Lee KH. Geographical Variations and Trends in Major Cancer Incidences throughout Korea during 1999-2013. 2018;50(4):1281–1293.).* Even in case of studies that denote the same research objective and data, differences in incidence may occur; further, AEGIS has often been able to estimate intermediate results.

Supplementary Figure S1: County-level age-adjusted geographical variation in incidence and mortality of major cancers in Korea.

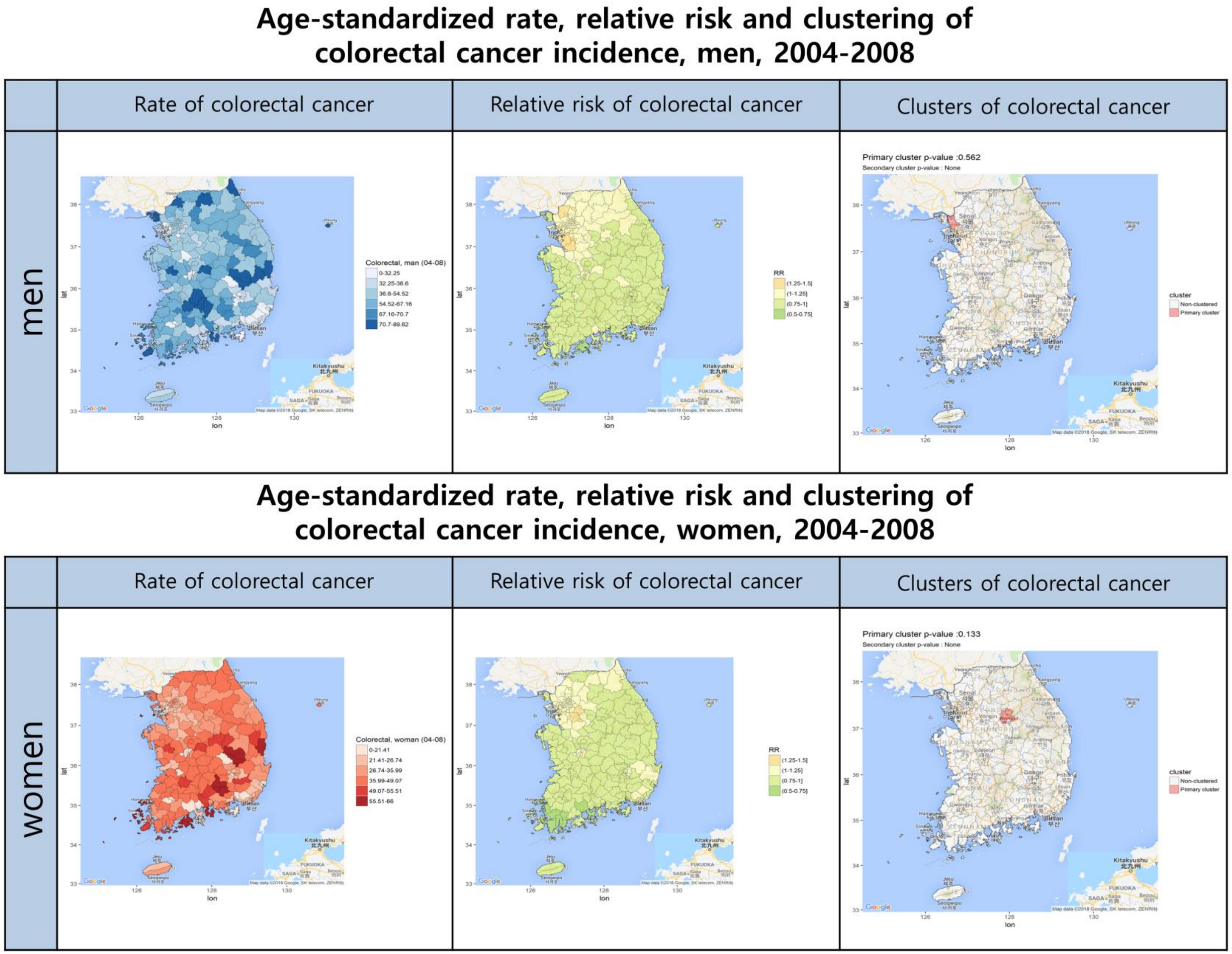

Age-standardized rate, relative risk and clustering of colorectal cancer incidence, men, 2009-2013

	Rate of colorectal cancer	Relative risk of colorectal cancer	Clusters of colorectal cancer
men	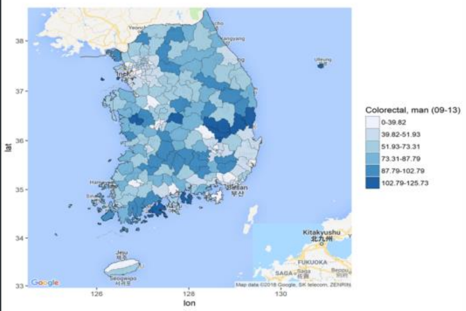	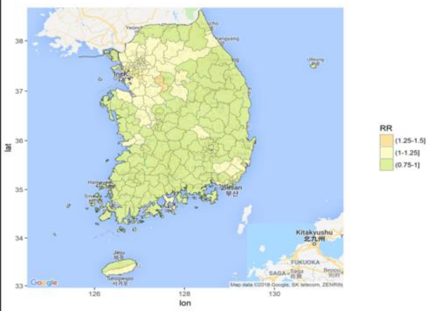	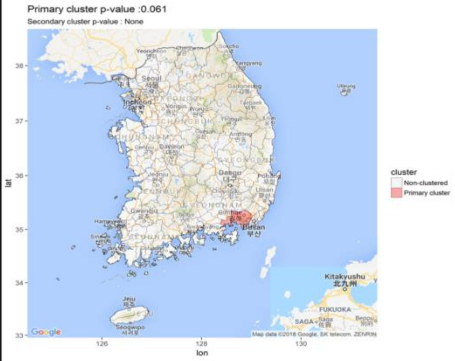

Age-standardized rate, relative risk and clustering of colorectal cancer incidence, women, 2009-2013

	Rate of colorectal cancer	Relative risk of colorectal cancer	Clusters of colorectal cancer
women	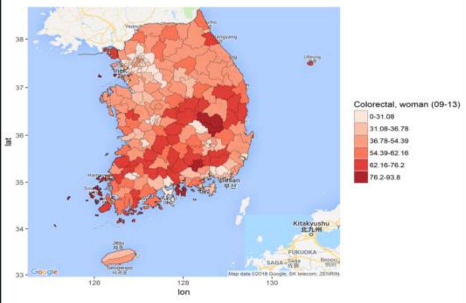	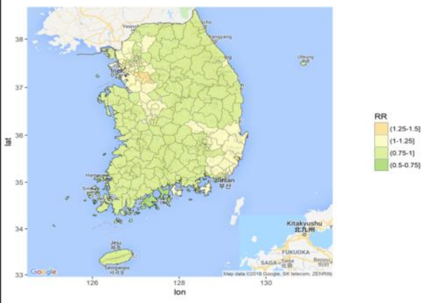	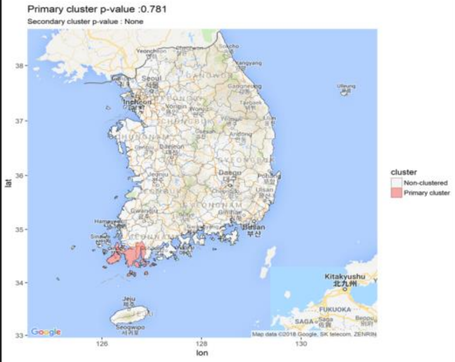

Age-standardized rate, relative risk and clustering of colorectal cancer mortality, men, 2004-2008

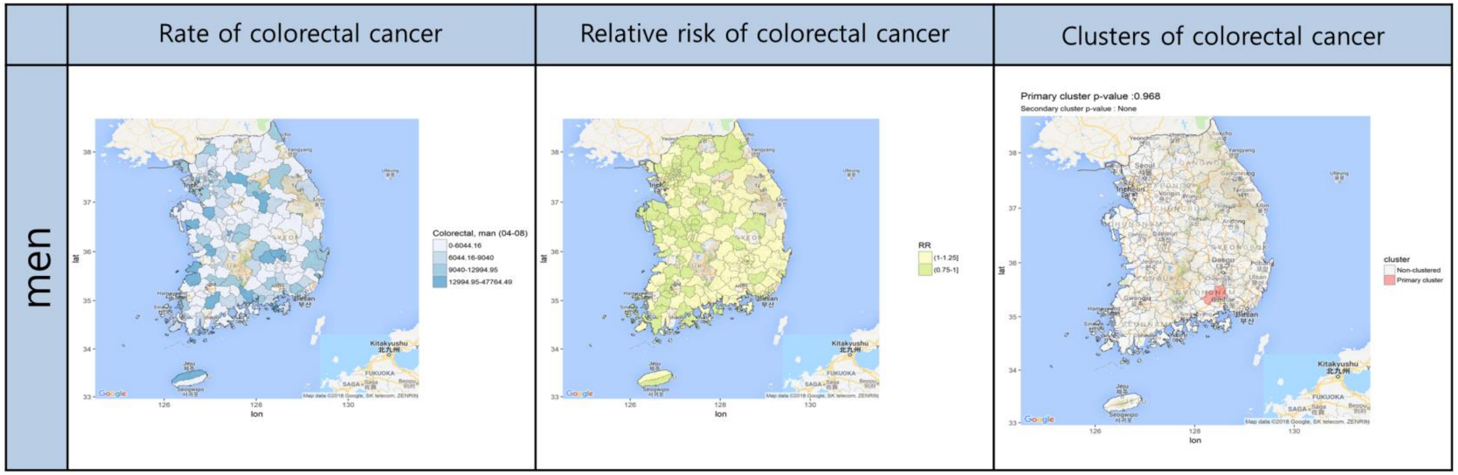

Age-standardized rate, relative risk and clustering of colorectal cancer mortality, women, 2004-2008

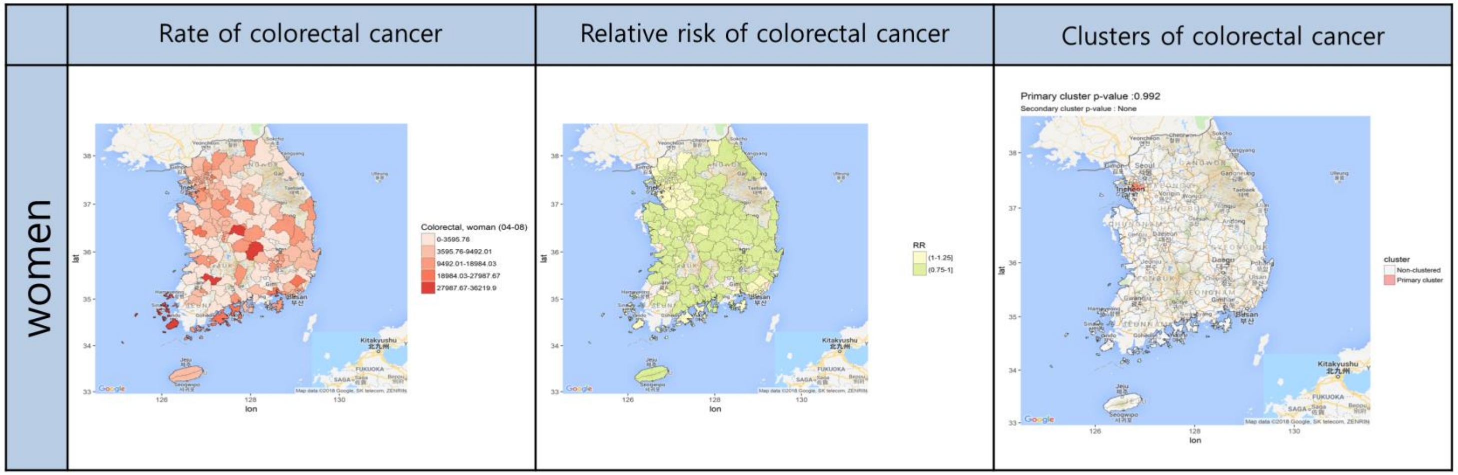

Age-standardized rate, relative risk and clustering of liver cancer incidence, men, 2004-2008

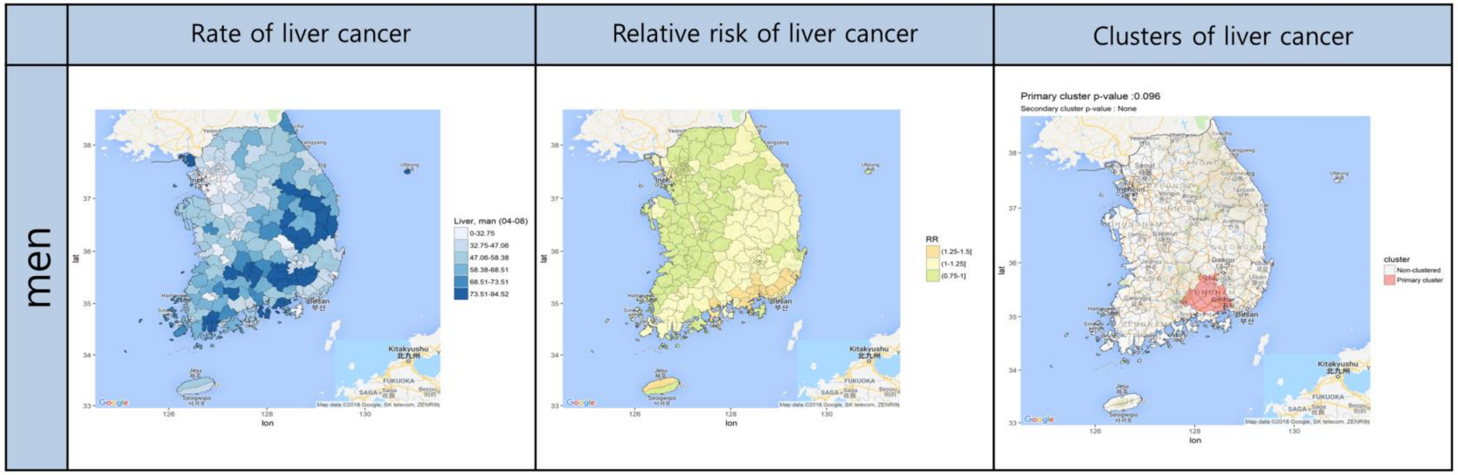

Age-standardized rate, relative risk and clustering of liver cancer incidence, women, 2004-2008

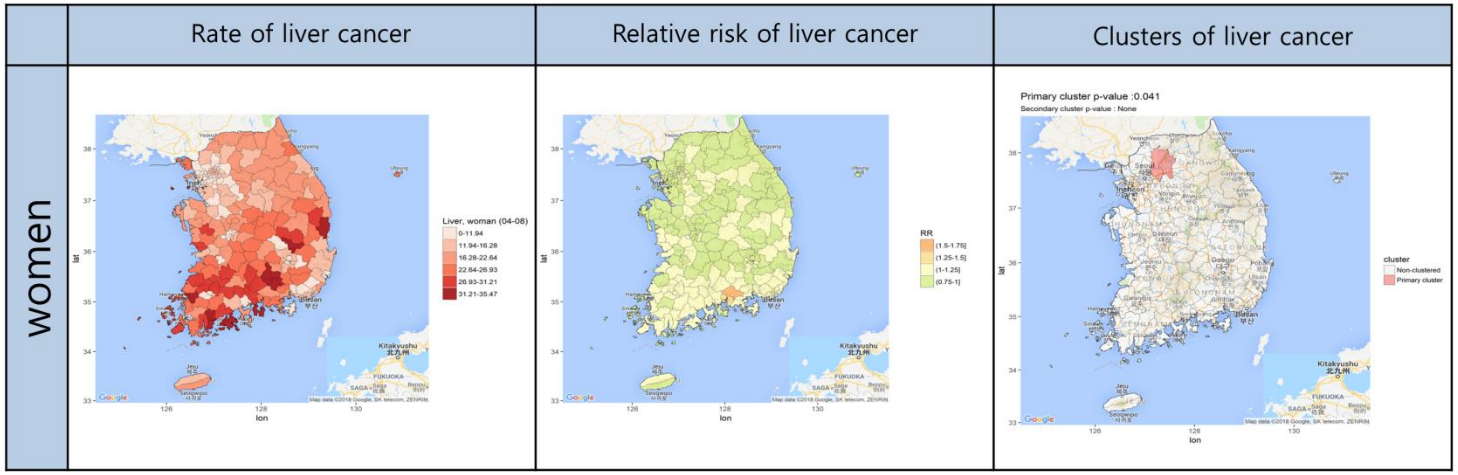

Age-standardized rate, relative risk and clustering of liver cancer incidence, men, 2009-2013

	Rate of liver cancer	Relative risk of liver cancer	Clusters of liver cancer
men	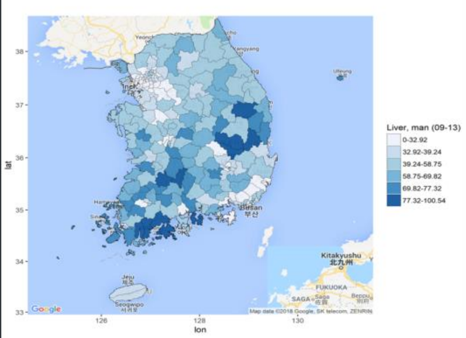	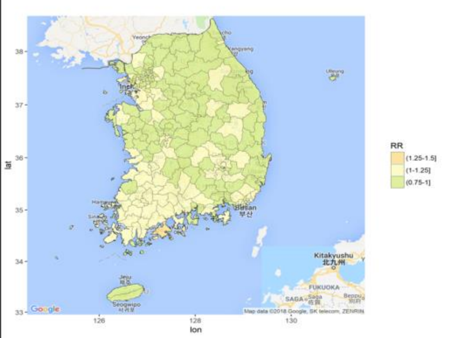	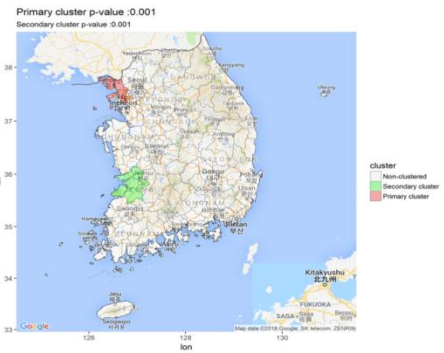

Age-standardized rate, relative risk and clustering of liver cancer incidence, women, 2009-2013

	Rate of liver cancer	Relative risk of liver cancer	Clusters of liver cancer
women	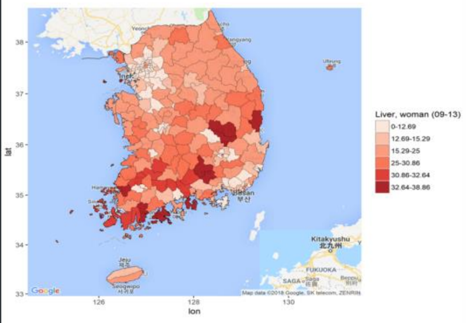	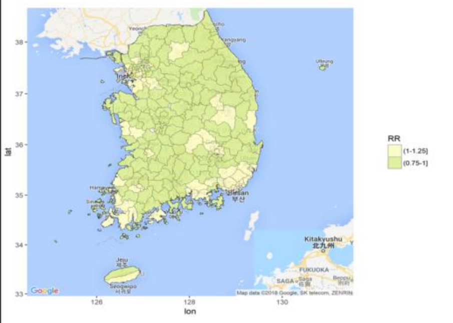	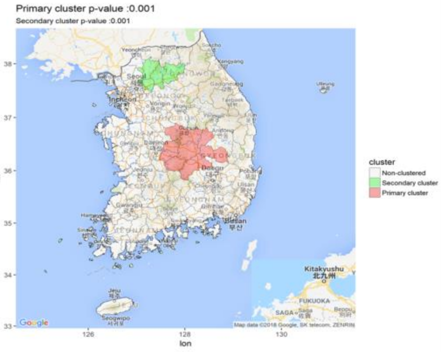

Age-standardized rate, relative risk and clustering of liver cancer mortality, men, 2004-2008

	Rate of liver cancer	Relative risk of liver cancer	Clusters of liver cancer
men	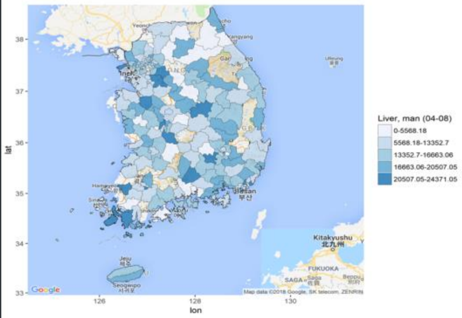	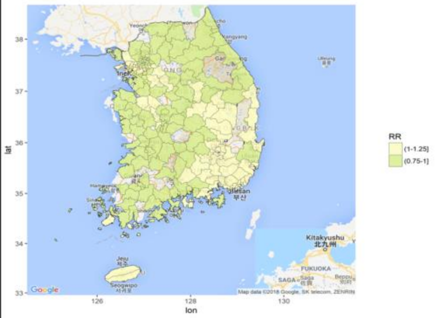	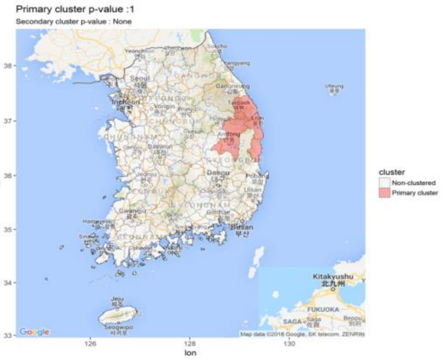

Age-standardized rate, relative risk and clustering of liver cancer mortality, women, 2004-2008

	Rate of liver cancer	Relative risk of liver cancer	Clusters of liver cancer
women	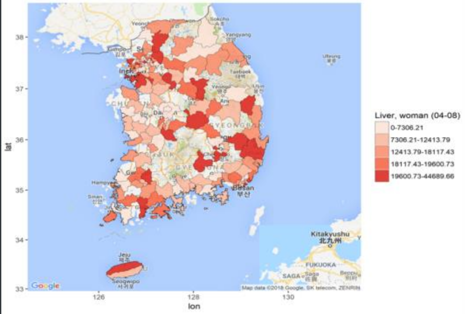	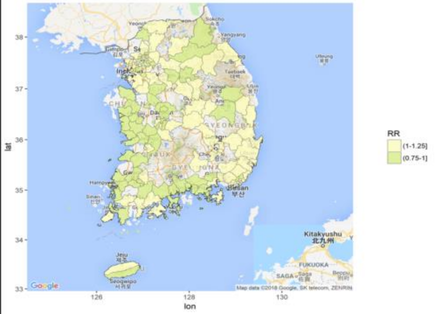	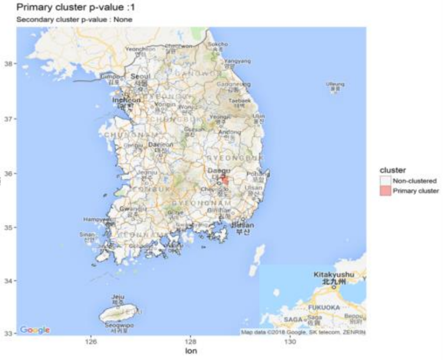

Age-standardized rate, relative risk and clustering of lung cancer incidence, men, 2004-2008

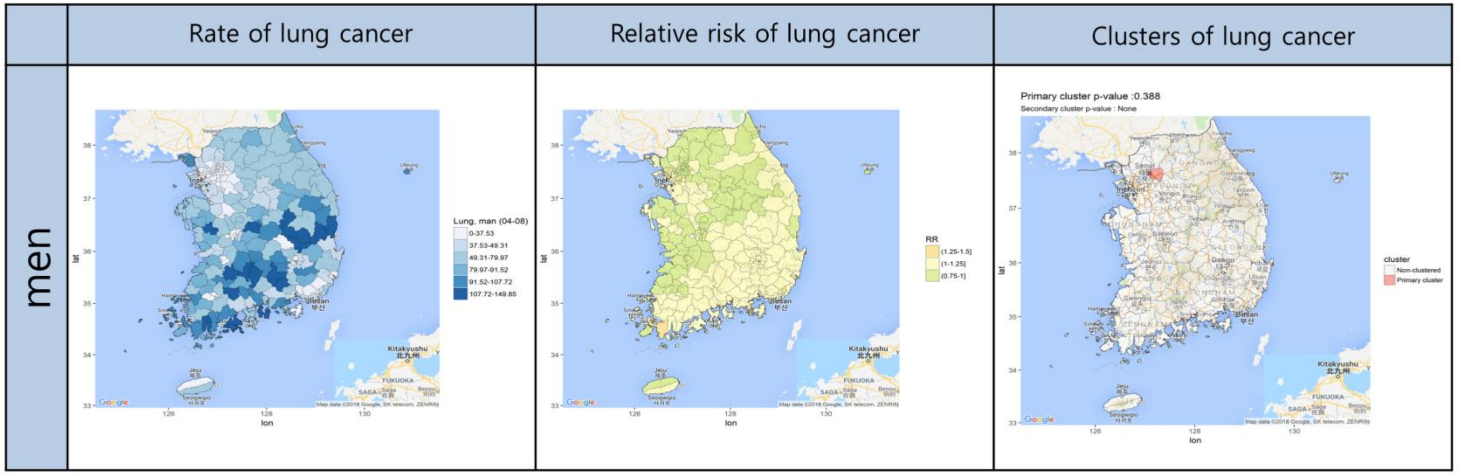

Age-standardized rate, relative risk and clustering of lung cancer incidence, women, 2004-2008

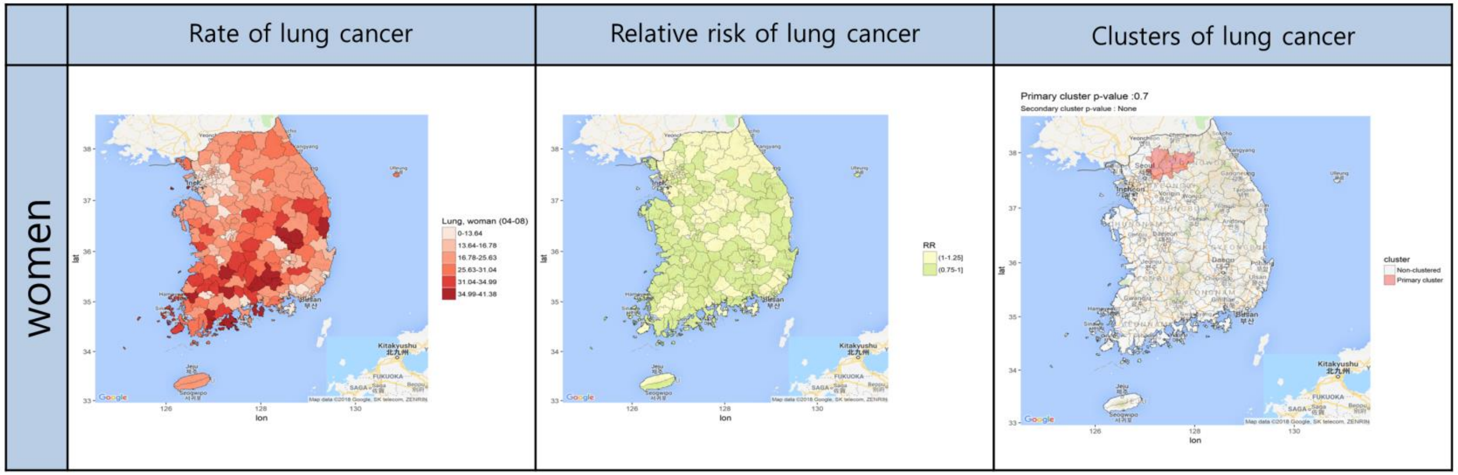

Age-standardized rate, relative risk and clustering of lung cancer incidence, men, 2009-2013

	Rate of lung cancer	Relative risk of lung cancer	Clusters of lung cancer
men	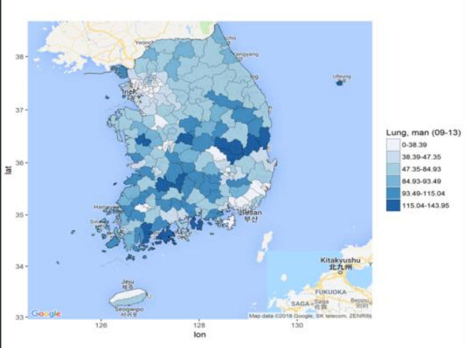	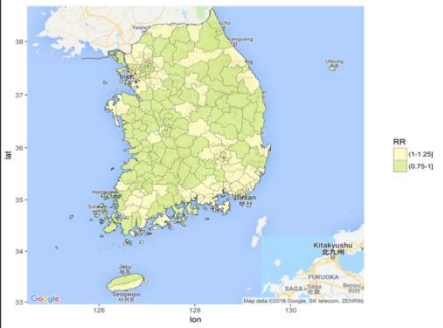	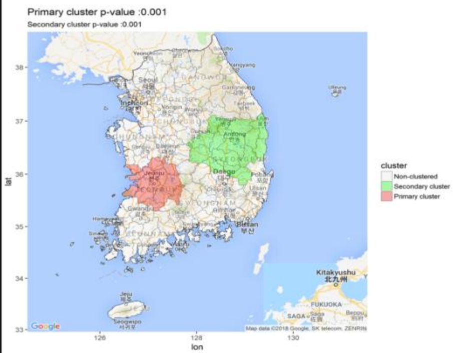

Age-standardized rate, relative risk and clustering of lung cancer incidence, women, 2009-2013

	Rate of lung cancer	Relative risk of lung cancer	Clusters of lung cancer
women	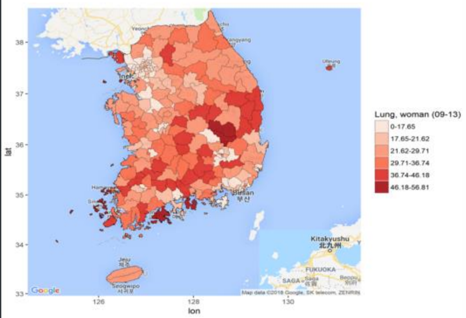	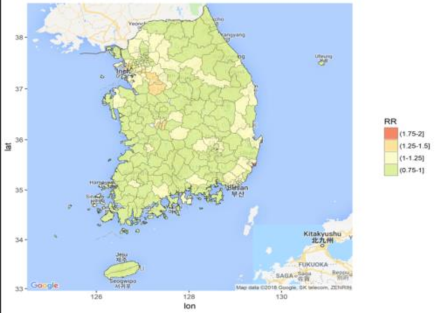	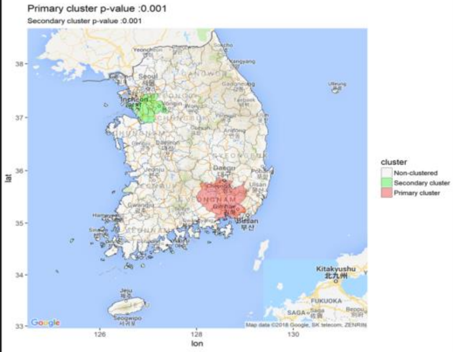

Age-standardized rate, relative risk and clustering of lung cancer mortality, men, 2004-2008

	Rate of lung cancer	Relative risk of lung cancer	Clusters of lung cancer
men	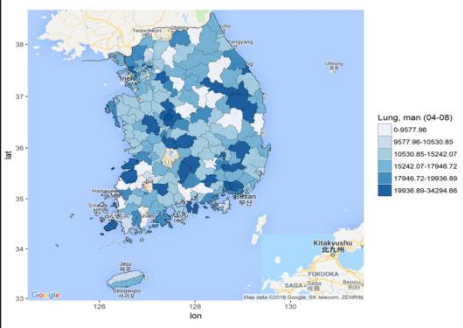	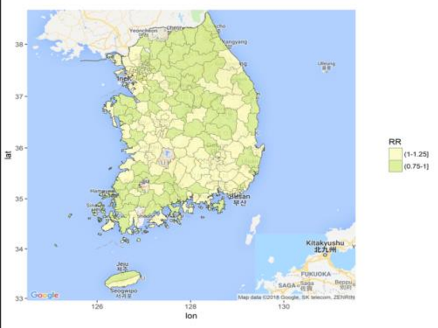	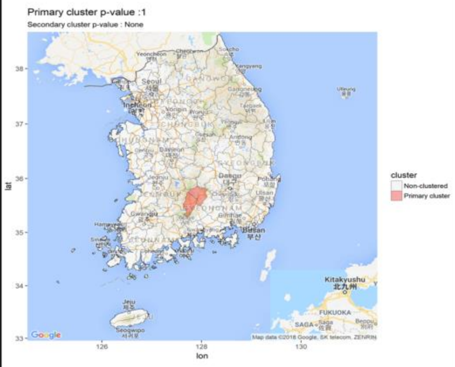

Age-standardized rate, relative risk and clustering of lung cancer mortality, women, 2004-2008

	Rate of lung cancer	Relative risk of lung cancer	Clusters of lung cancer
women	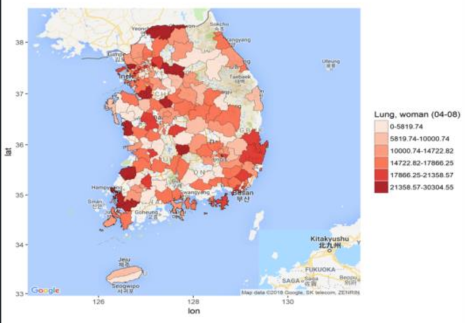	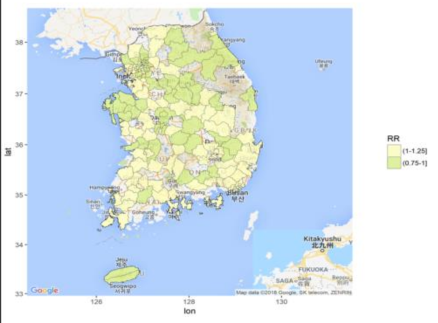	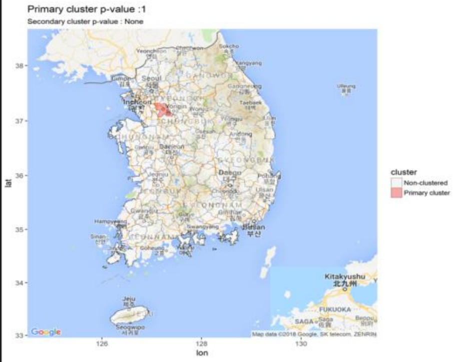

Age-standardized rate, relative risk and clustering of stomach cancer incidence, men, 2004-2008

	Rate of stomach cancer	Relative risk of stomach cancer	Clusters of stomach cancer
men	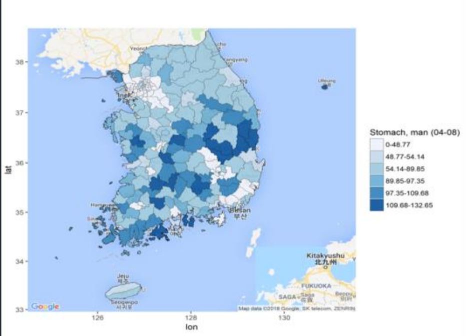	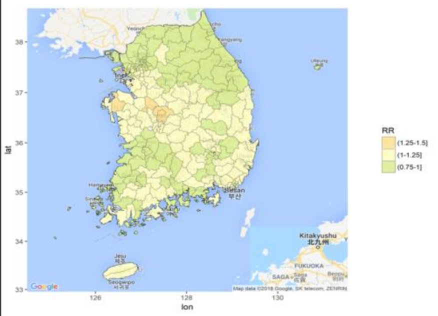	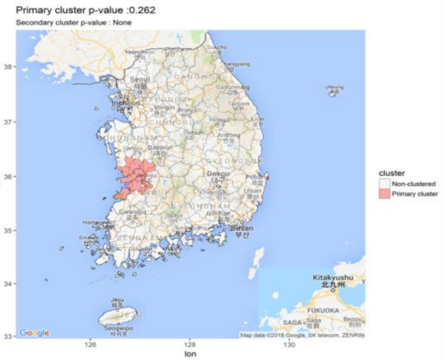

Age-standardized rate, relative risk and clustering of stomach cancer incidence, women, 2004-2008

	Rate of stomach cancer	Relative risk of stomach cancer	Clusters of stomach cancer
women	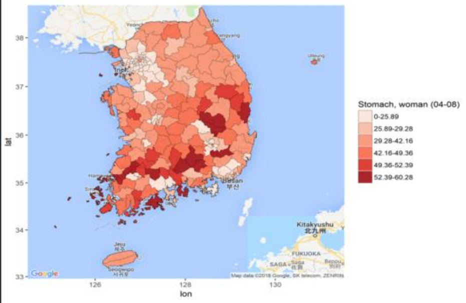	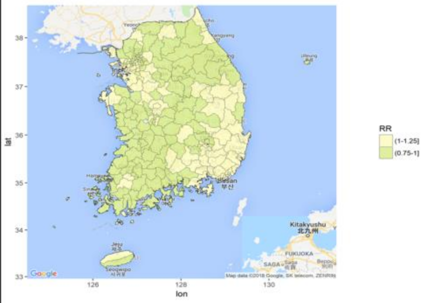	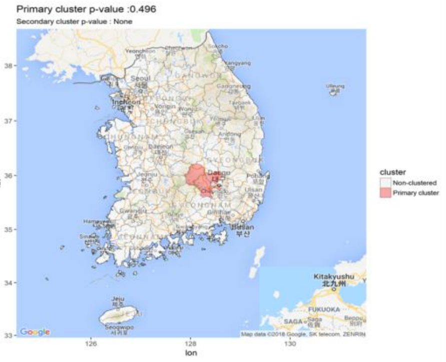

Age-standardized rate, relative risk and clustering of stomach cancer incidence, men, 2009-2013

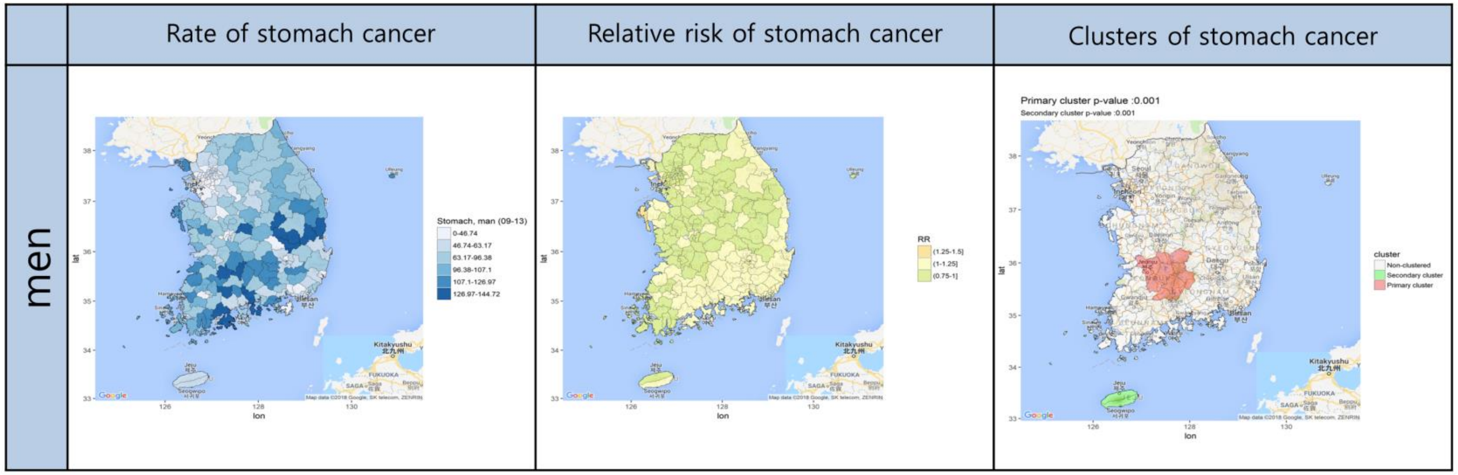

Age-standardized rate, relative risk and clustering of stomach cancer incidence, women, 2009-2013

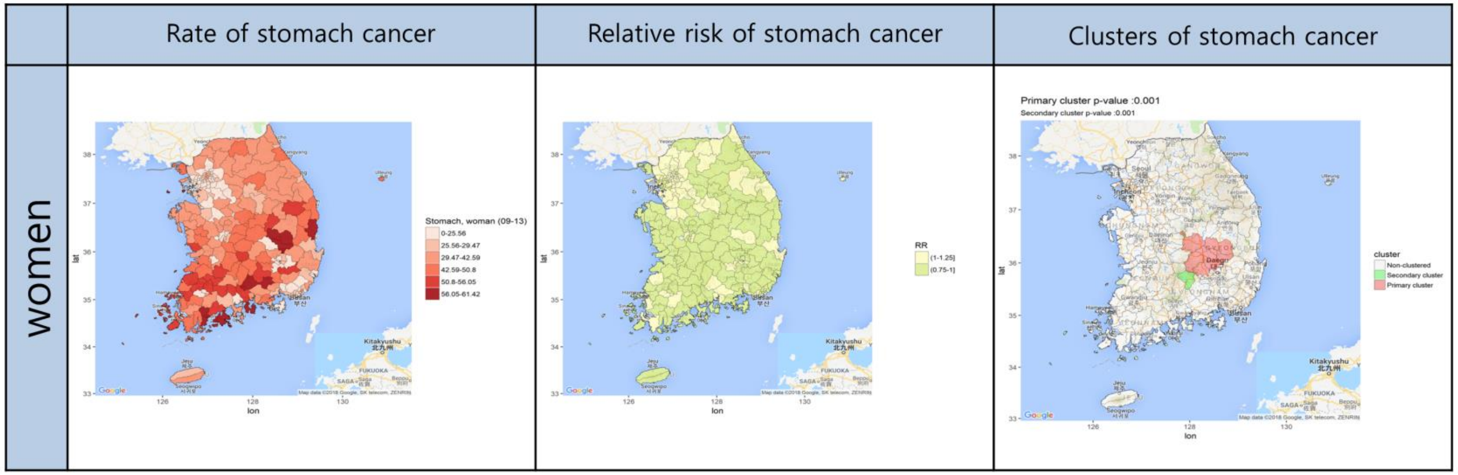

Age-standardized rate, relative risk and clustering of stomach cancer mortality, men, 2004-2008

men

Rate of stomach cancer

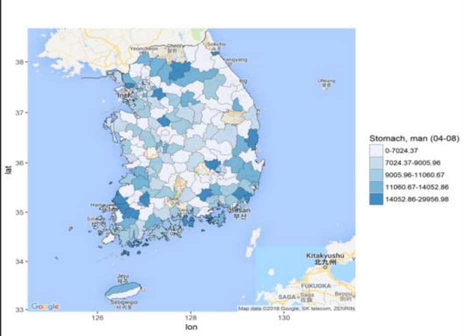

Relative risk of stomach cancer

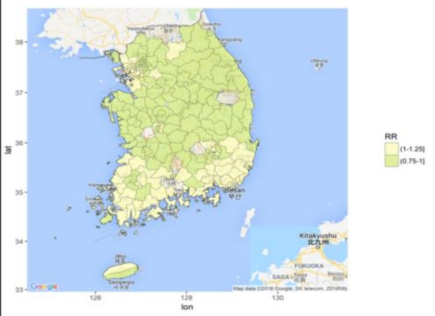

Clusters of stomach cancer

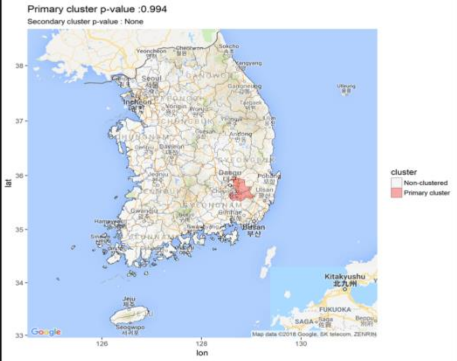

Age-standardized rate, relative risk and clustering of stomach cancer mortality, women, 2004-2008

women

Rate of stomach cancer

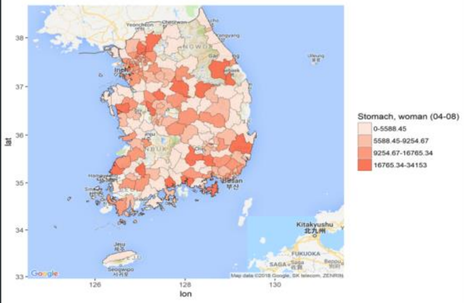

Relative risk of stomach cancer

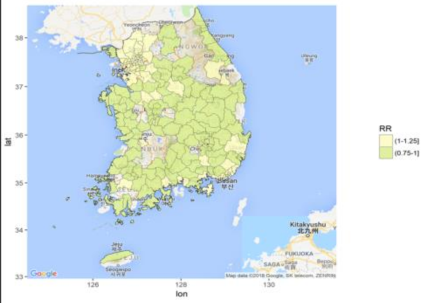

Clusters of stomach cancer

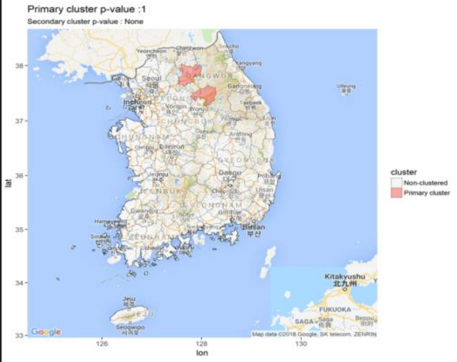

Age-standardized rate, relative risk and clustering of thyroid cancer incidence, men, 2004-2008

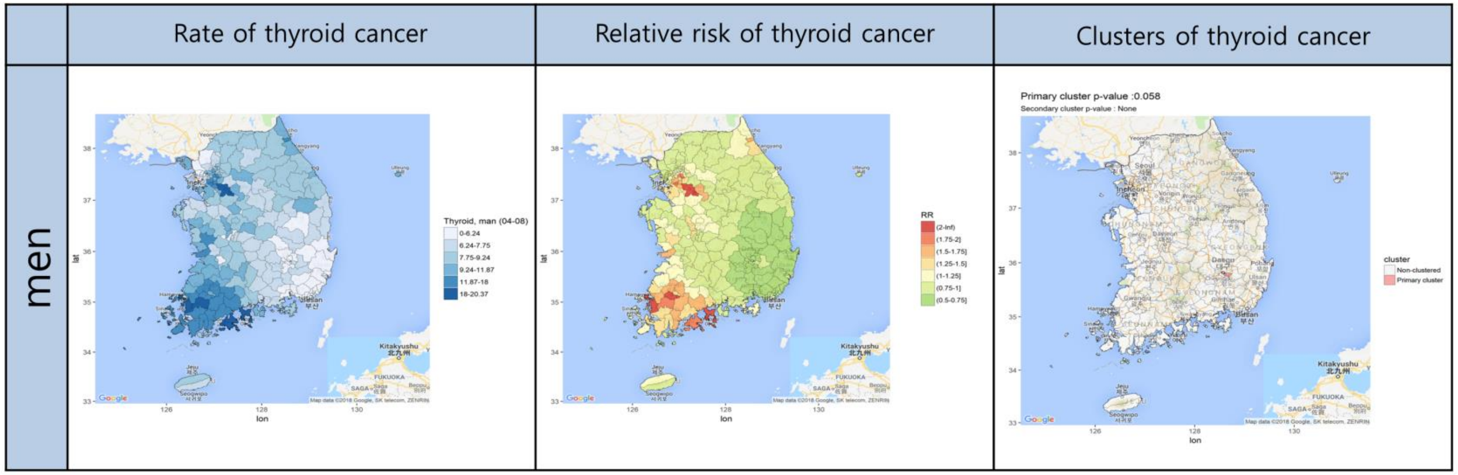

Age-standardized rate, relative risk and clustering of thyroid cancer incidence, women, 2004-2008

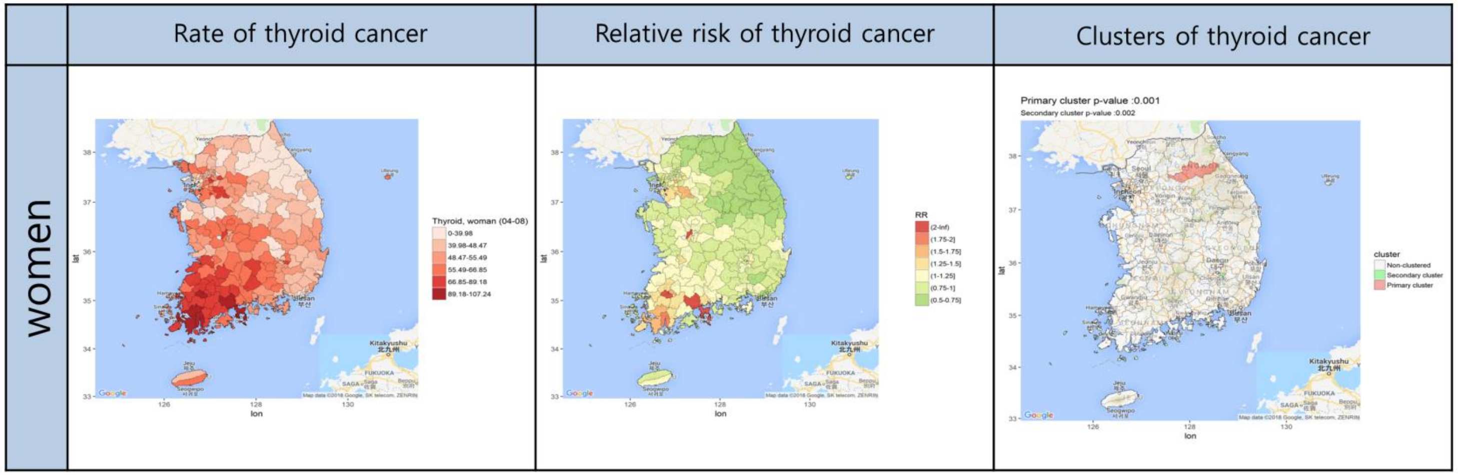

Age-standardized rate, relative risk and clustering of thyroid cancer incidence, men, 2009-2013

	Rate of thyroid cancer	Relative risk of thyroid cancer	Clusters of thyroid cancer
men	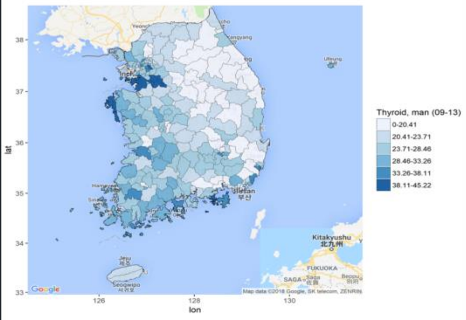	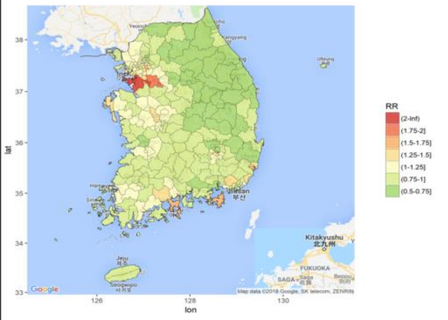	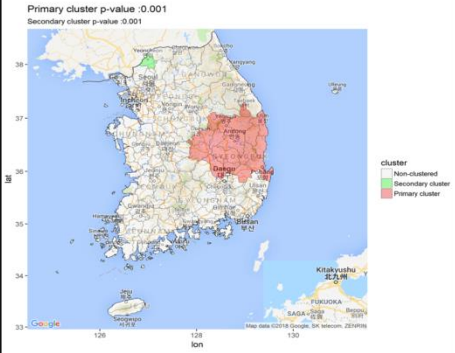

Age-standardized rate, relative risk and clustering of thyroid cancer incidence, women, 2009-2013

	Rate of thyroid cancer	Relative risk of thyroid cancer	Clusters of thyroid cancer
women	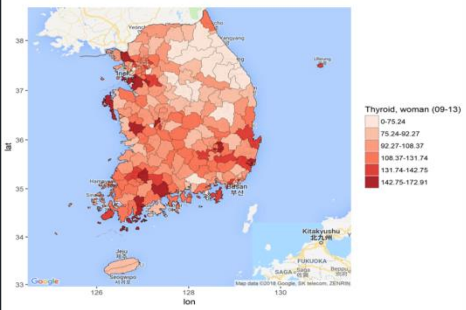	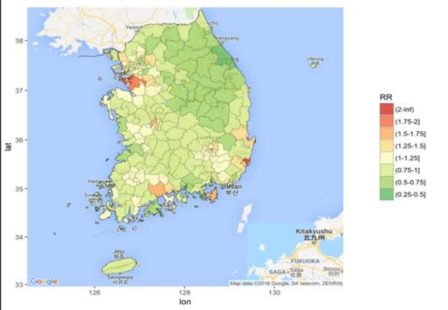	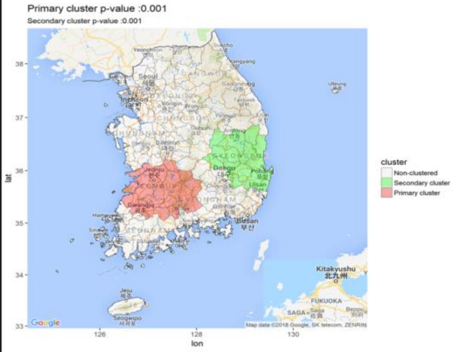

Age-standardized rate, relative risk and clustering of breast cancer incidence, women, 2004-2008

	Rate of breast cancer	Relative risk of breast cancer	Clusters of breast cancer
women	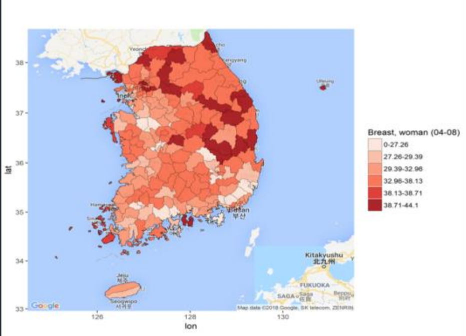	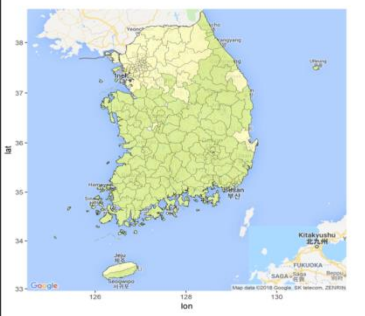	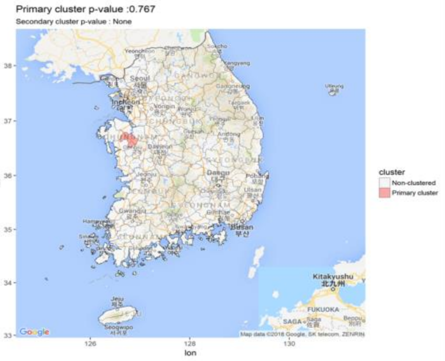

Age-standardized rate, relative risk and clustering of breast cancer incidence, women, 2009-2013

	Rate of breast cancer	Relative risk of breast cancer	Clusters of breast cancer
women	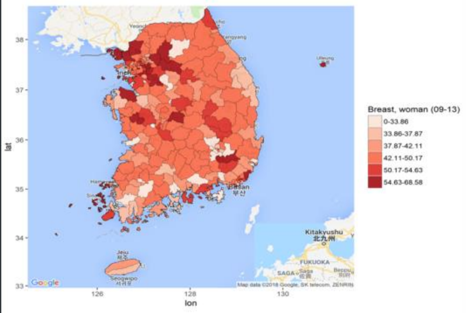	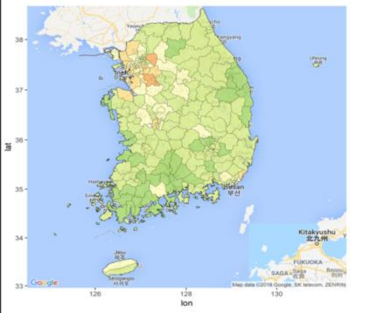	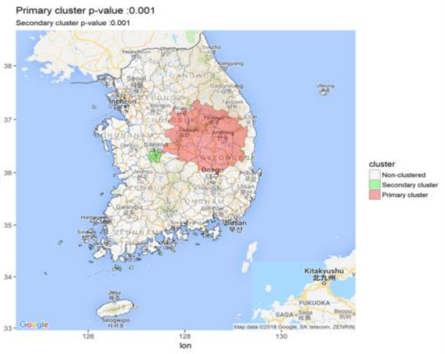

Age-standardized rate, relative risk and clustering of breast cancer mortality, women, 2004-2008

	Rate of breast cancer	Relative risk of breast cancer	Clusters of breast cancer
women	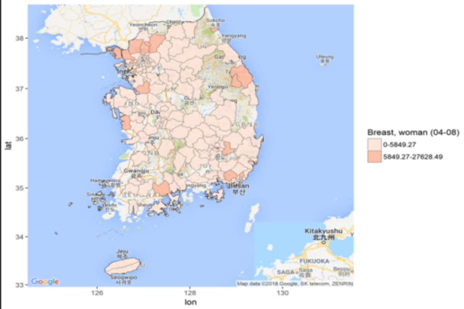	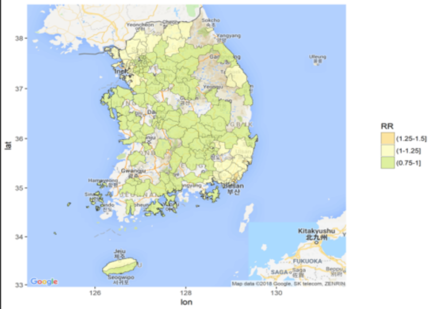	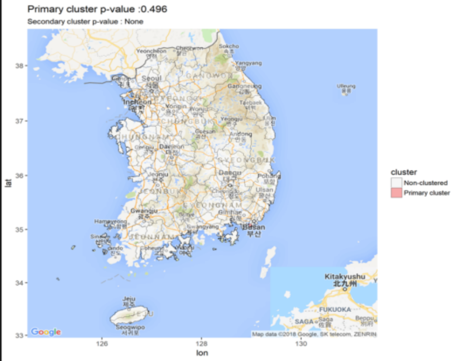

Age-standardized rate, relative risk and clustering of prostate cancer incidence, men, 2004-2008

<table>
<tr><th></th><th>Rate of prostate cancer</th><th>Relative risk of prostate cancer</th><th>Clusters of prostate cancer</th></tr>
<tr><td>men</td><td>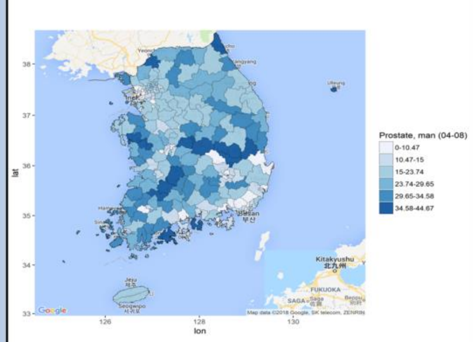
</td><td>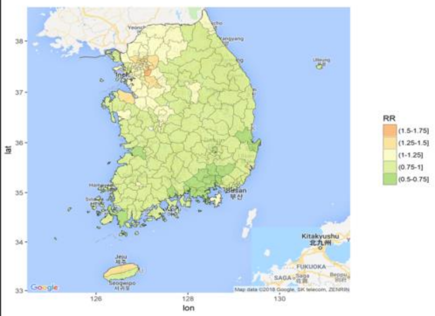
</td><td>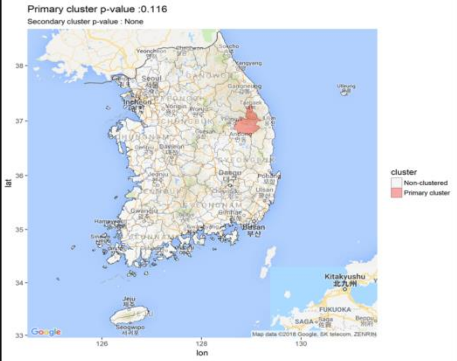
</td></tr>
</table>

Age-standardized rate, relative risk and clustering of prostate cancer incidence, men, 2009-2013

<table>
<tr><th></th><th>Rate of prostate cancer</th><th>Relative risk of prostate cancer</th><th>Clusters of prostate cancer</th></tr>
<tr><td>men</td><td>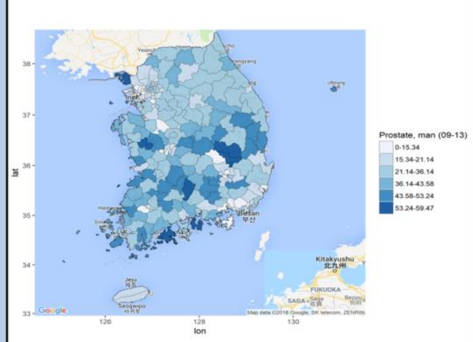
</td><td>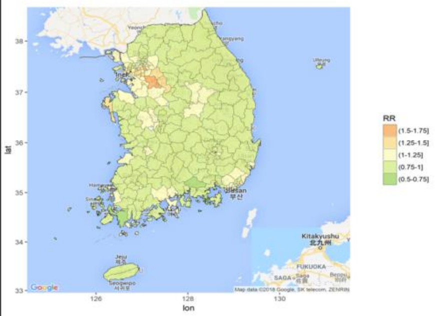
</td><td>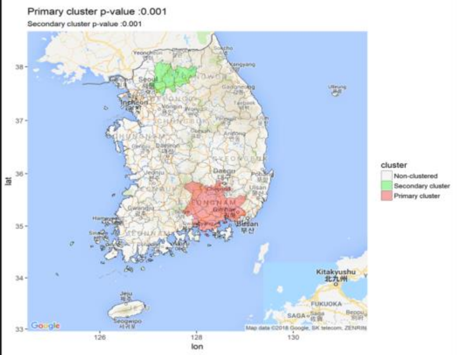
</td></tr>
</table>

Age-standardized rate, relative risk and clustering of prostate cancer mortality, men, 2004-2008

	Rate of prostate cancer	Relative risk of prostate cancer	Clusters of prostate cancer
men	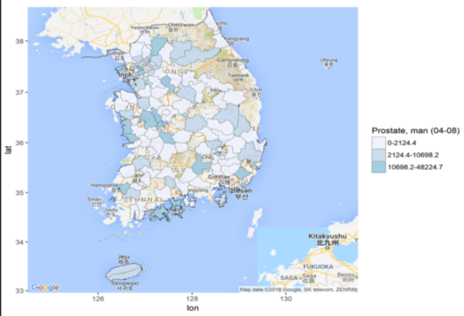	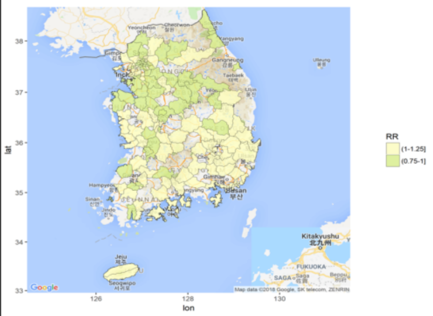	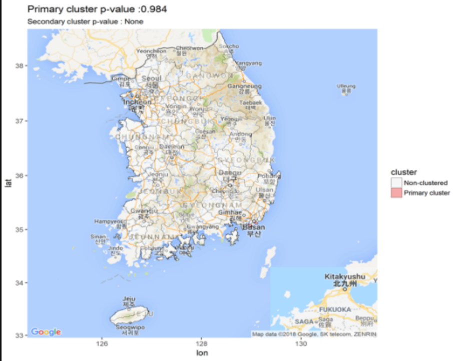

International Journal of
Environmental Research and Public Health

MDPI

Supplementary Figure S2: Comparison of GADM-level 2 major cancer age-standardized incidence rate from AEGIS and major cancer age-standardized incidence rate from NCC in Korea.

1. Comparison of age-standardized rate (red line) with 95% credible interval (red color) estimated from AEGIS and age-standardized rate reported from NCC (blue line) for major cancer between 2004 and 2008 in men.

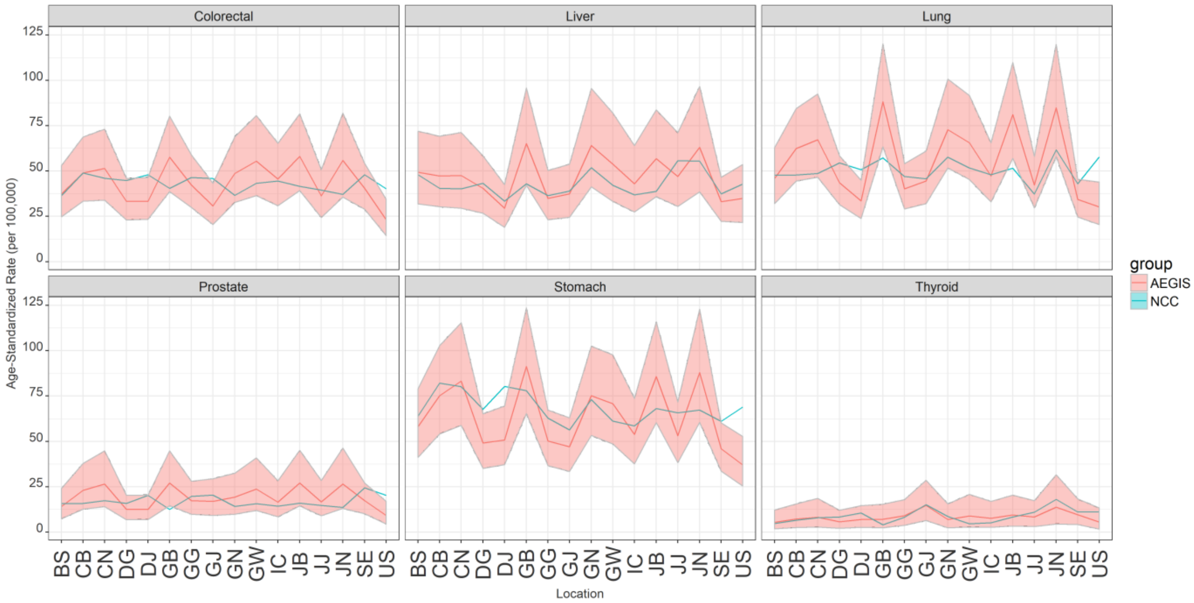

2. Comparison of age-standardized rate (red line) with 95% credible interval (red color) estimated from AEGIS and age-standardized rate reported from NCC (blue line) for major cancer between 2004 and 2008 in women.

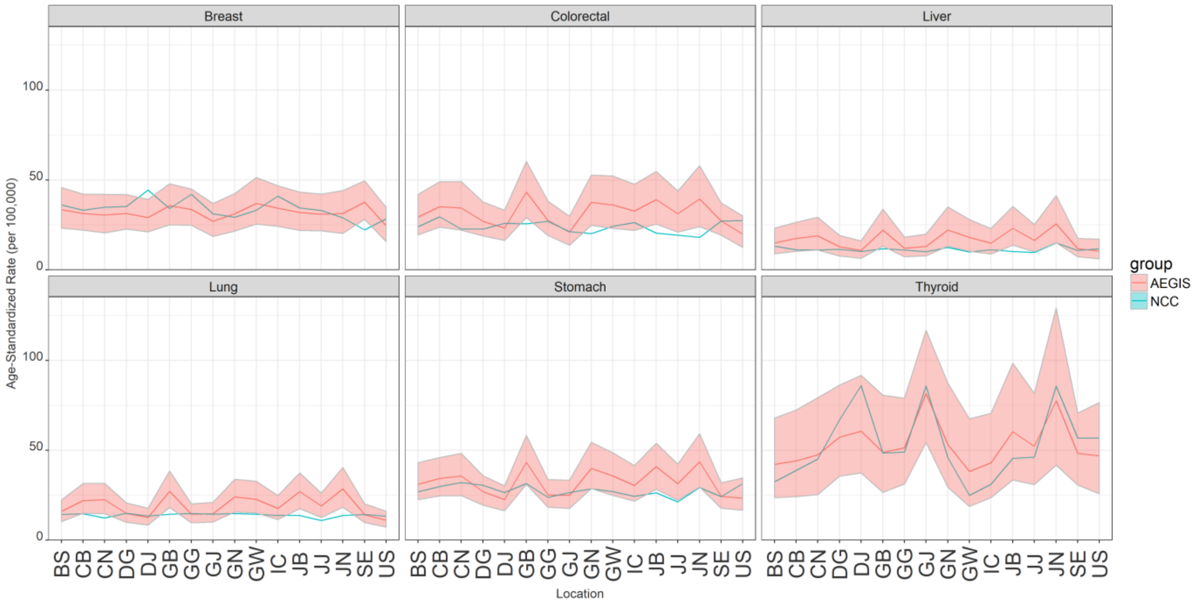

3. Comparison of age-standardized rate (red line) with 95% credible interval (red color) estimated from AEGIS and age-standardized rate reported from NCC (blue line) for major cancer between 2009 and 2013 in men.

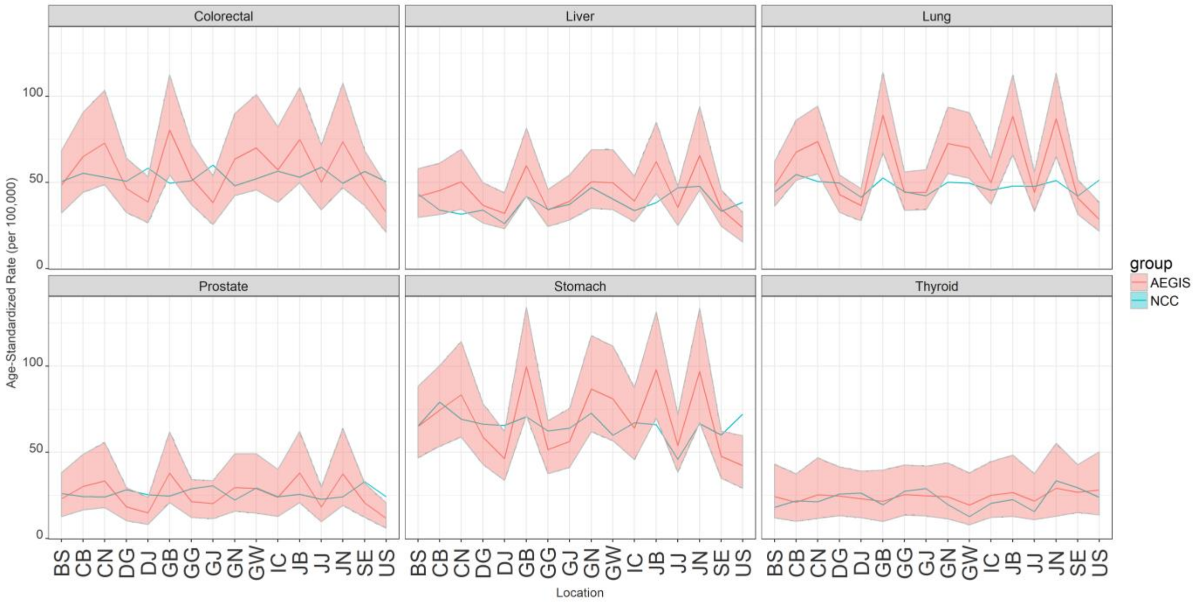

4. Comparison of age-standardized rate (red line) with 95% credible interval (red color) estimated from AEGIS and age-standardized rate reported from NCC (blue line) for major cancer between 2009 and 2013 in women.

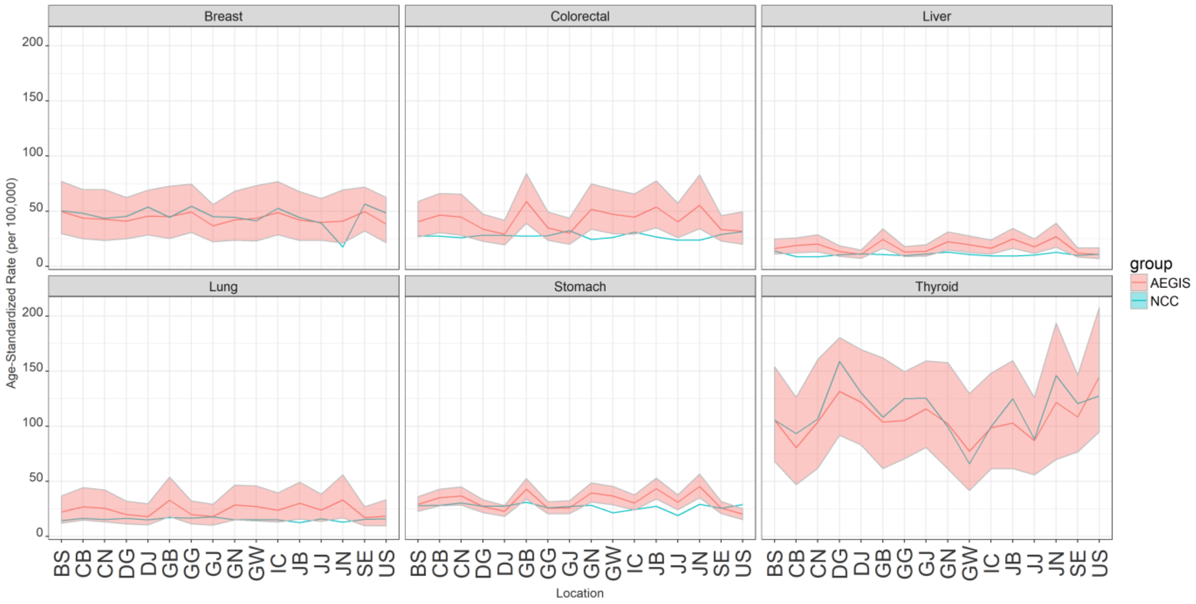

BS: Busan; CB: Chungcheongbuk-do; CN: Chungcheongnam-do; DG: Daegu; DJ: Daejeon; GB: Gyeongsangbuk-do; GG: Gyeonggi-do; GJ: Gwangju; GN: Gyeongsangnam-do; GW: Gangwon-do; IC: Incheon; JB: Jeollabuk-do; JJ: Jeju; JN:Jeollanam-do; SE: Seoul; US: Ulsan

Supplementary Figure S3: Disease mapping and clustering for regional differences in hospitalization rates due to heart-related diseases per 1,000 people in the United States from 2008 to 2010 (age and sex adjusted).

We also designed a spatial study involving a variety of heart diseases for further comparison with the previously published study 'Interactive Atlas of Heart Disease and Stroke' (https://www.cdc.gov/dhdsp/maps/atlas/index.htm) in US sources. The target cohorts were defined as the whole population in the database, and the outcome cohorts included patients hospitalized for stroke, acute myocardial infarction, cardiac dysfunction, coronary heart disease, heart failure, and heart disease, respectively, between 2008 and 2010.

1. Stroke

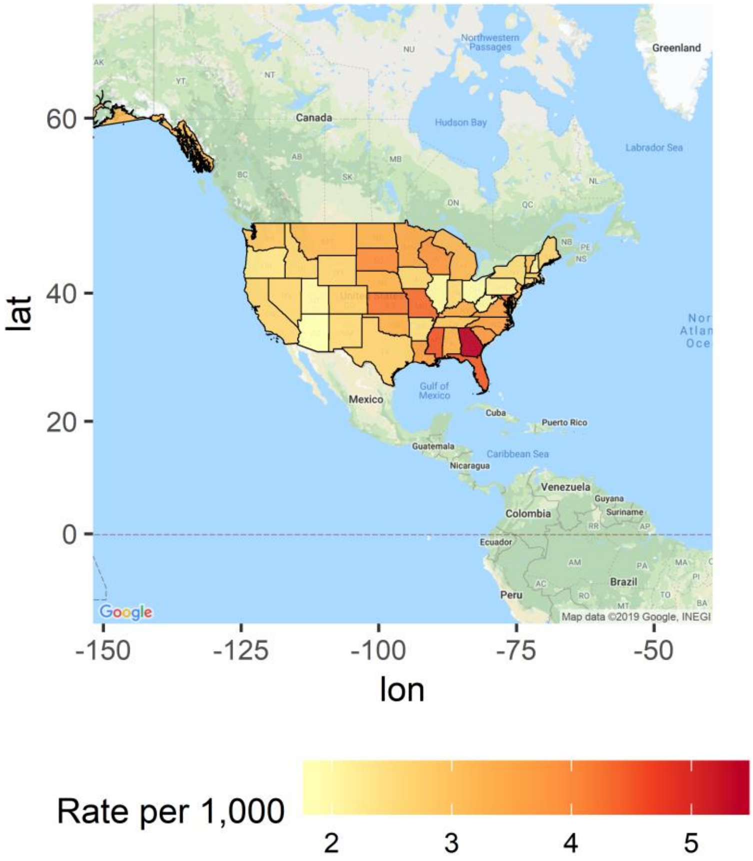
lat
lon
Rate per 1,000
2
3
4
5

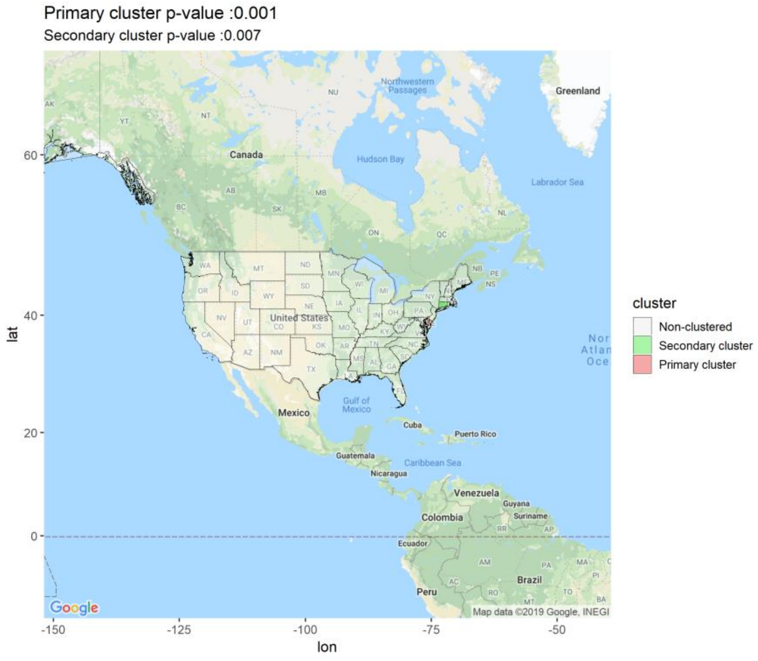
Primary cluster p-value :0.001
Secondary cluster p-value :0.007
lat
lon
cluster
Non-clustered
Secondary cluster
Primary cluster

2. Acute Myocardial Infarction

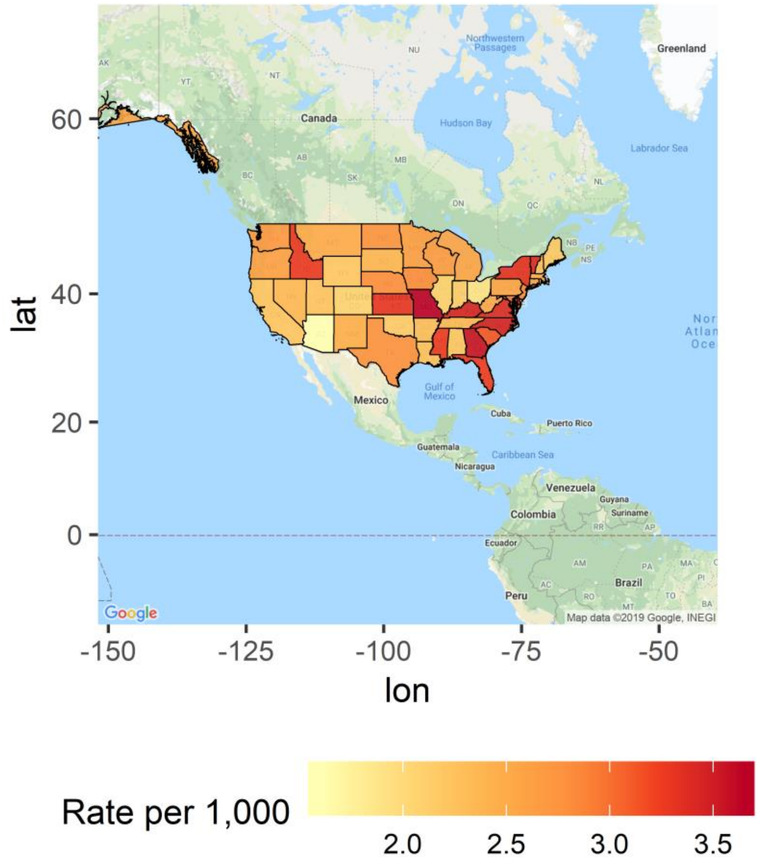

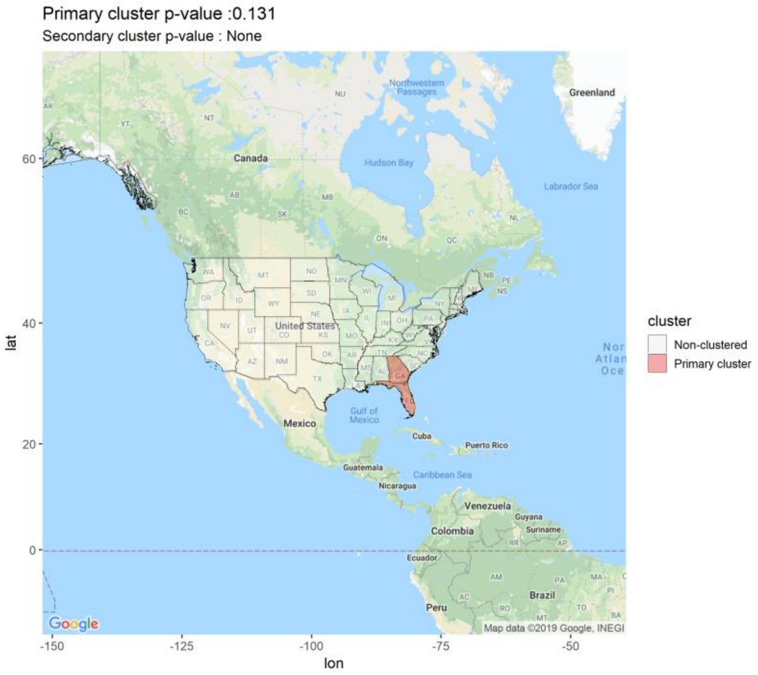

3. Cardiac Dysrhythmia

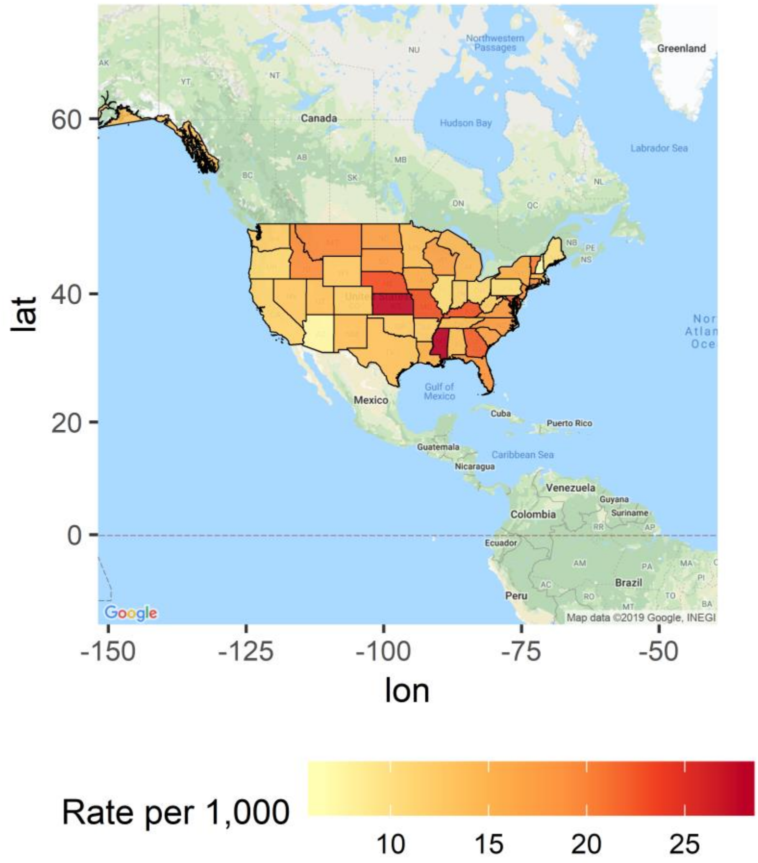

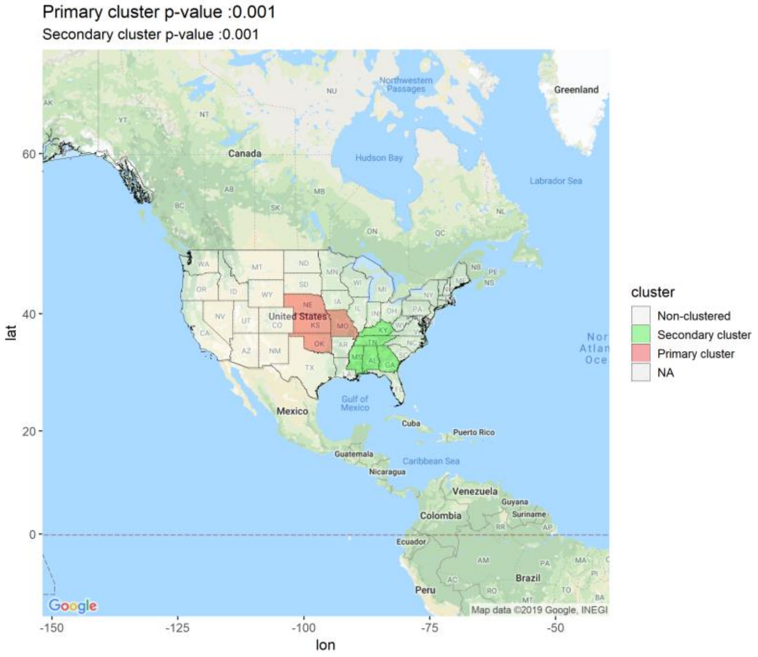

4. Coronary Heart Disease

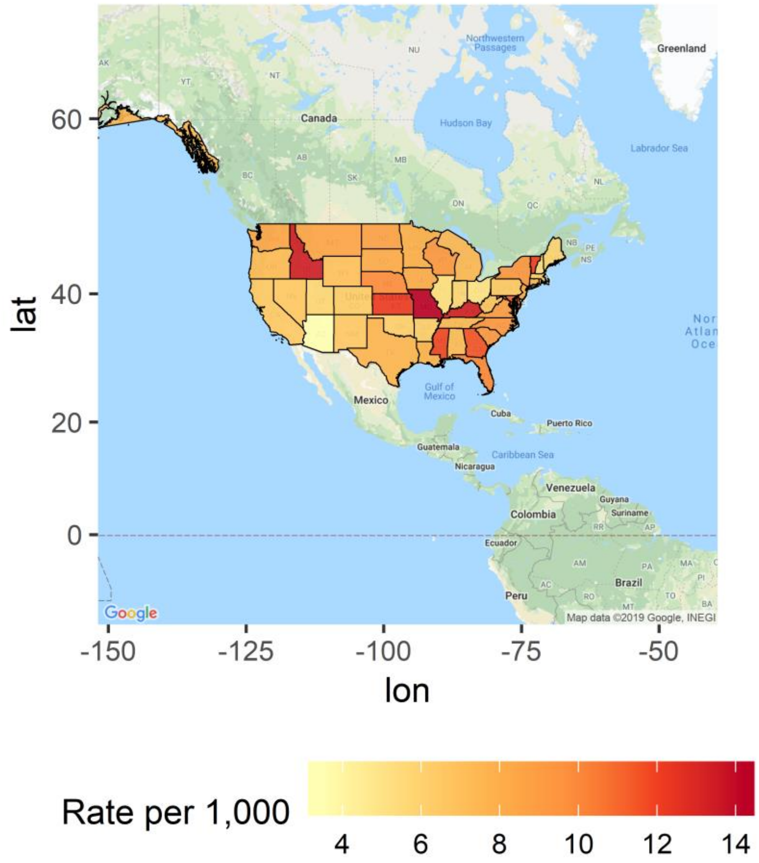

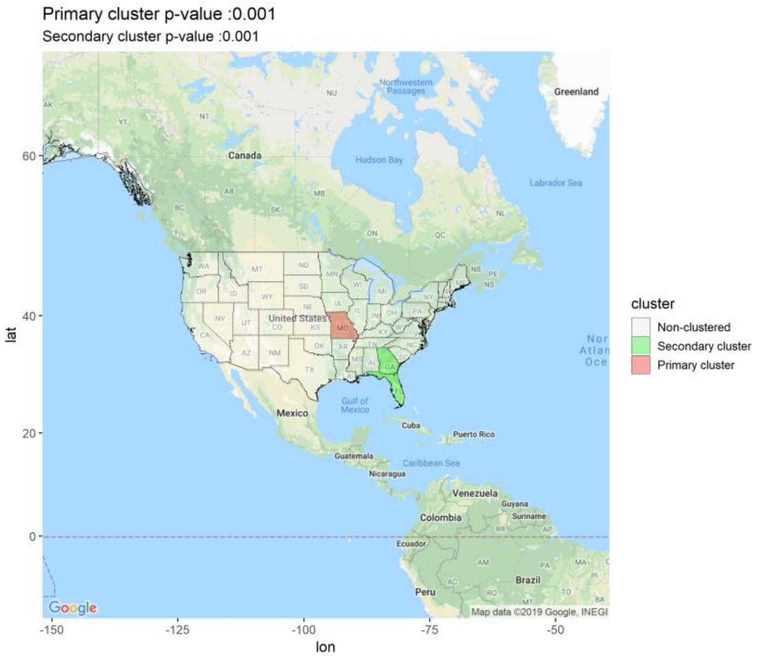

5. Heart Failure

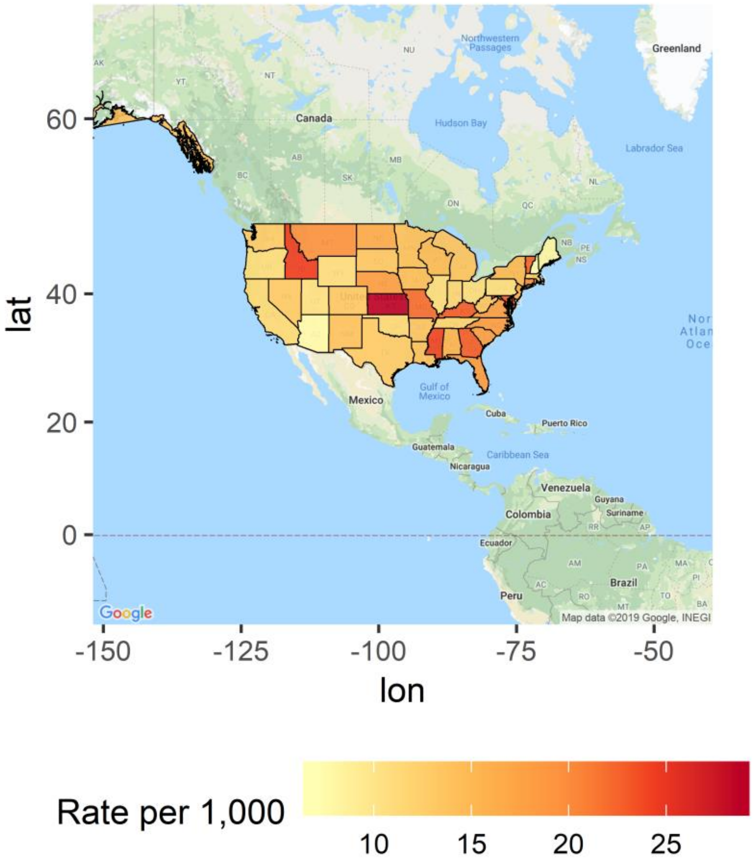

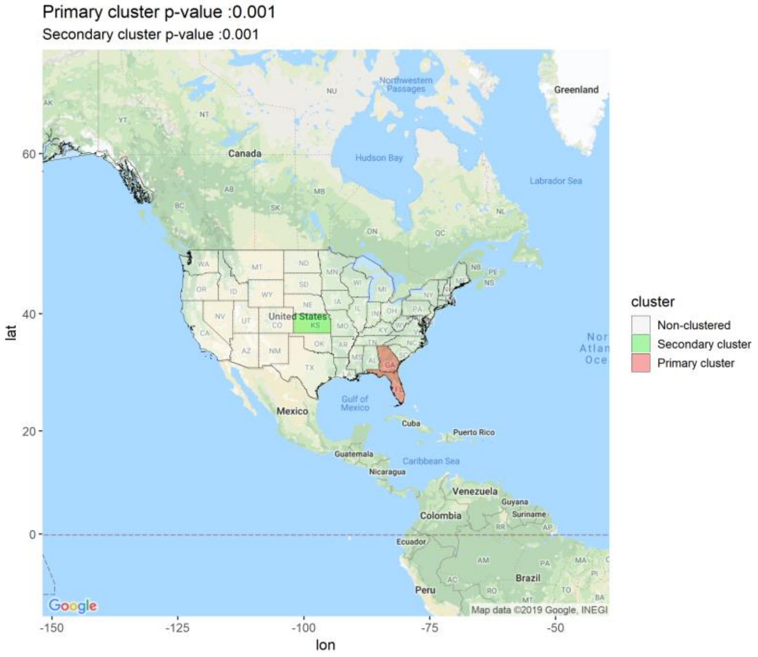

6. Heart disease

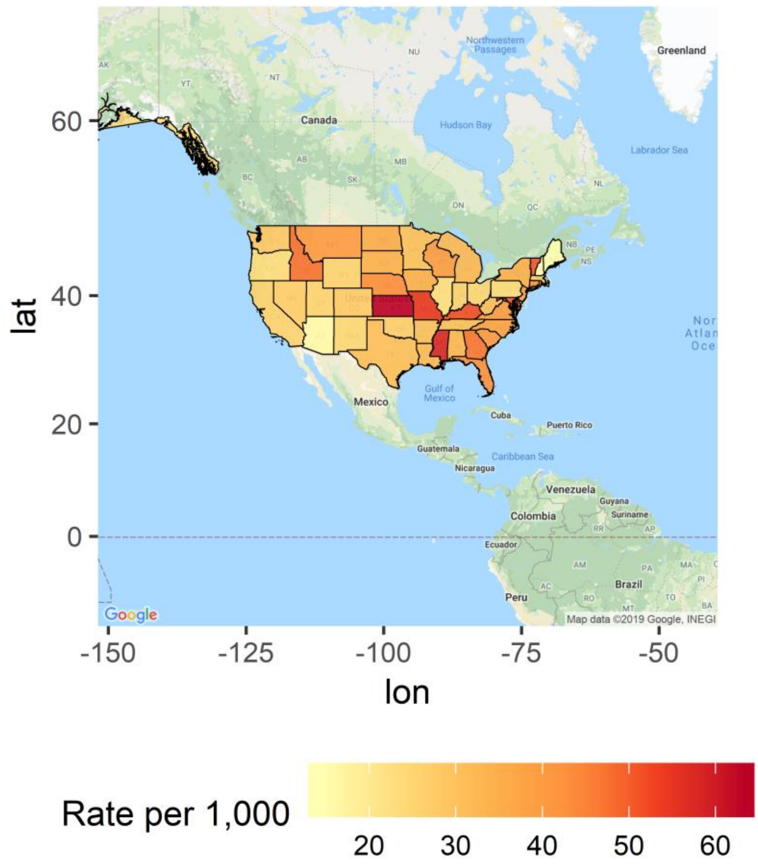
lat
60
40
20
0
-150
-125
-100
-75
-50
lon
Rate per 1,000
20
30
40
50
60
Canada
Hudson Bay
Greenland
Labrador Sea
Mexico
Gulf of Mexico
Cuba
Puerto Rico
Caribbean Sea
Venezuela
Colombia
Brazil
Peru
Map data ©2019 Google, INEGI

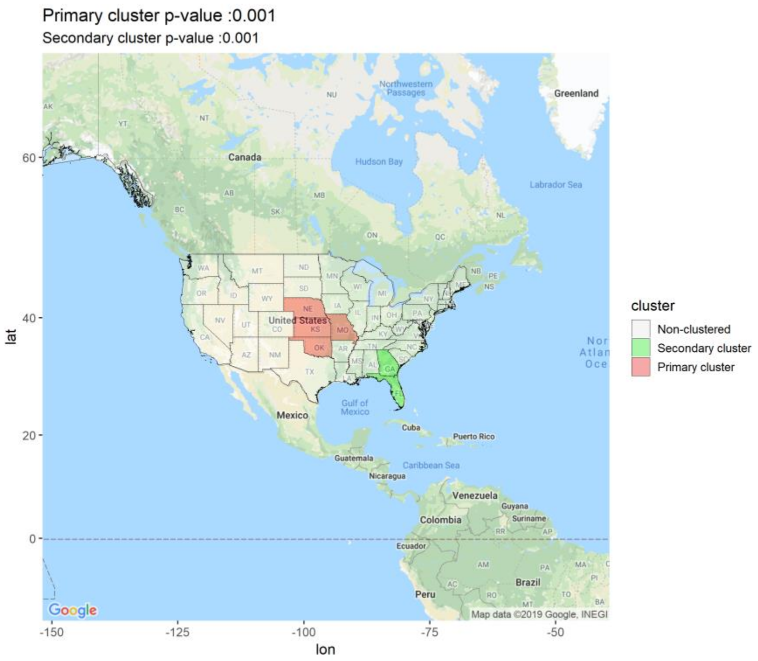
Primary cluster p-value :0.001
Secondary cluster p-value :0.001
lat
60
40
20
0
-150
-125
-100
-75
-50
lon
cluster
Non-clustered
Secondary cluster
Primary cluster
United States
Canada
Hudson Bay
Greenland
Mexico
Map data ©2019 Google, INEGI

Supplementary Information S1: Interactive web application

AEGIS interactive web application provides spatial analysis design by setting parameters in each function tabs on the left side (red box). Define the target cohort and outcome cohort to be analyzed from the population (green box). Combine two defined cohorts and GIS data, select options (including data handling options, method, and parameters; green box) for analysis and output the analysis results (blue box). The parameters provided through the interactive interface are shown in Figure A and are described below. It also provides a brief demonstration of the video format to help researchers who are new to AEGIS (https://youtu.be/tExqsZU7qYg).

WARNINGS: The results of AEGIS depend on the used healthcare database. AEGIS proportionate units of analysis and supports small area estimation techniques to prevent sampled location bias or false positive, but it does not guarantee nationwide results. Therefore, the results of AEGIS should be interpreted considering the geographical/medical rationale along with the characteristics of the data used.

A. *DB connection panel:* To configure the CDM server connection, set the server address, user name, password, database management systems, and CDM database schema.

B. *Cohorts:* Spatial analysis research design by setting user parameters. (1) select the target cohort and outcome cohort defined from the ATLAS; (2) set range of date to analyze with select windows parameter; (3) adjust age and gender differences between regions through age and gender adjustment parameters; (4) set the time-at-risk parameter for observing the outcome from the index date; and (5) select the country of study from the country list (total = 254 countries).

C. *Disease mapping:* From the settings designed in Cohorts tab, it is a panel for disease map visualization. Choose an administrative level with an administrative level parameter, and determine how to draw a disease map (Count of the target cohort (n), Proportion, Standardized Incidence Ratio, Bayesian mapping). Finally, set the entire title of the map and the title of the legend.

D. *Clustering:* In this tab, researchers can choose statistical methods (Local Indicators of Spatial Association, Kulldorff method) to detect disease clusters in which disease occurrences are concentrated. Visualize the results according to the selected method.

AEGIS can download and run packages from R with the following code:

```
install.packages("devtools")

devtools::install_github("cran/raster", ref="2.6-7")

devtools::install_github("ohdsi/DatabaseConnector")

devtools::install_github("ohdsi/SqlRender")

devtools::install_github("ohdsi/aegis")

AEGIS::AEGIS()
```

Figure A. Illustration of the AEGIS that performs spatial epidemiology analysis using observational health data based on OMOP-CDM.

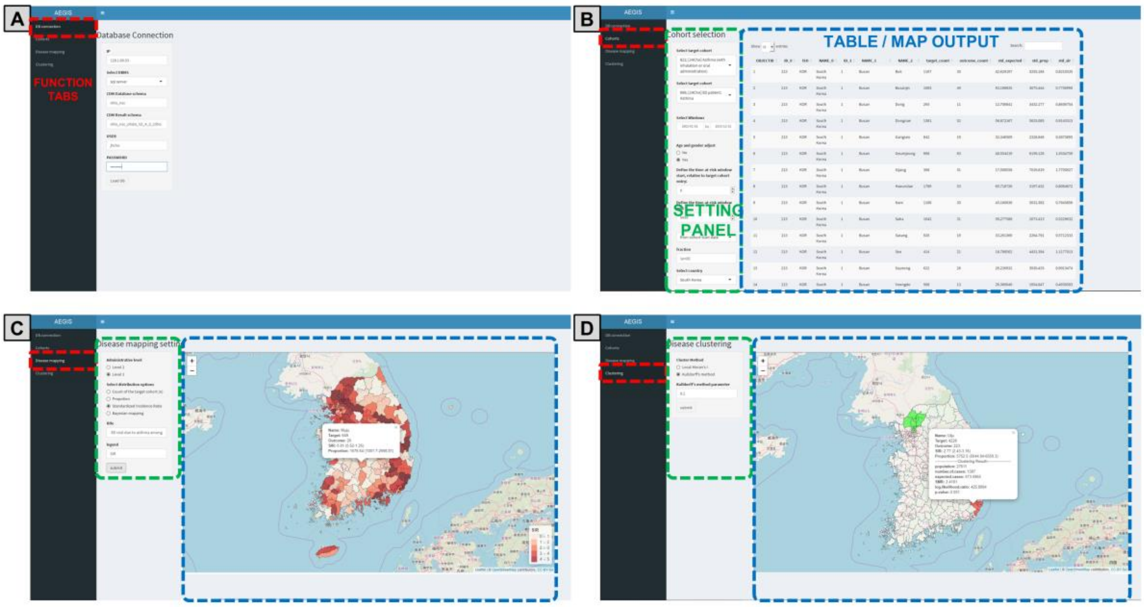
A
AEGIS
Database Connection
FUNCTION TABS
B
AEGIS
Cohort selection
TABLE / MAP OUTPUT
SETTING PANEL
C
AEGIS
Disease mapping setti
D
AEGIS
Disease clustering
